# Genome sequencing and analysis uncover the regulatory elements involved in the development and oil biosynthesis of *Pongamia pinnata* (L.) – A potential biodiesel feedstock

**DOI:** 10.3389/fpls.2022.747783

**Published:** 2022-08-25

**Authors:** Rachapudi Venkata Sreeharsha, Shalini Mudalkar, Attipalli Ramachandra Reddy

**Affiliations:** ^1^Department of Plant Sciences, University of Hyderabad, Hyderabad, India; ^2^Department of Life Sciences, Chhatrapati Shahu Ji Maharaj University, Kanpur, India; ^3^Department of Tree Breeding and Improvement, Forest College and Research Institute (FCRI), Hyderabad, India

**Keywords:** *Millettia pinnata*, genome, lipid biosynthesis, biodiesel, Illumina sequencing, karanjin, *Pongamia pinnata*

## Abstract

Due to rapid industrialization, the consumption of petro-products has increased, while fossil fuel resources have been gradually depleted. There has been a resurgence of interest in plant-derived biofuels as a sustainable alternative to fossil fuels for the purpose of reducing greenhouse gas emissions. *Pongamia pinnata* L., which is also known as *Millettia pinnata* is an oil-yielding, leguminous tree with a large and complex genome. Despite its multiple industrial applications, this orphan tree species has inconsistent yields and a limited understanding of its functional genomics. We assessed physiological and morphological characteristics of five high-yielding pongamia accessions and deduced important yield descriptors. Furthermore, we sequenced the genome of this potential biofuel feedstock using Illumina HiSeq, NextSeq, and MiSeq platforms to generate paired-end reads. Around 173 million processed reads amounting to 65.2 Gb were assembled into a 685 Mb genome, with a gap rate of 0.02%. The sequenced scaffolds were used to identify 30,000 gene models, 406,385 Simple-Sequence-Repeat (SSR) markers, and 43.6% of repetitive sequences. We further analyzed the structural information of genes belonging to certain key metabolic pathways, including lipid metabolism, photosynthesis, circadian rhythms, plant-pathogen interactions, and karanjin biosynthesis, all of which are commercially significant for pongamia. A total of 2,219 scaffolds corresponding to 29 transcription factor families provided valuable information about gene regulation in pongamia. Similarity studies and phylogenetic analysis revealed a monophyletic group of Fabaceae members wherein pongamia out-grouped from *Glycine max* and *Cajanus cajan*, revealing its unique ability to synthesize oil for biodiesel. This study is the first step toward completing the genome sequence of this imminent biofuel tree species. Further attempts at re-sequencing with different read chemistry will certainly improve the genetic resources at the chromosome level and accelerate the molecular breeding programs.

## Introduction

The unprecedented increase in atmospheric CO_2_ concentrations, along with the depletion of fossil fuels, is increasing the demand for a reliable supply of carbon-neutral fuels. The use of biological carbon fixation to generate energy is an ideal complement to rapidly dwindling fossil fuel reserves. However, uninterrupted feedstock supplies have become a major impediment to sustainable biofuels and bioenergy production. Bioenergy/biofuel trees can offset 1,000 to 2,000 metric tons of carbon (C) per year through carbon sequestration and energy cropping (Babin et al., 2021). As a result, they meet a major portion of the world’s energy needs while also contributing to the reduction of CO_2_ levels in the atmosphere. Appropriate biofuel plant selection is contingent on the socioeconomic and environmental factors of agricultural practices. Among the several alternatives for producing biofuels and bioenergy, *Pongamia pinnata* L. has garnered widespread attention due to its diversified growth habitats and low-input agriculture. *Pongamia pinnata* (L.), alternatively known as *Millettia pinnata* (L.), is a member of the Fabaceae family that grows in a range of Indian tropical and subtropical marginal lands (Arote and Yeole, 2010; Sreeharsha et al., 2016). It is a semi-deciduous, nitrogen-fixing tree that grows to a height of 15–20 m with a broad canopy and is known for its drought and salinity tolerance. The dense network of pongamia lateral roots aids in soil erosion control *via* binding sand dunes. The seeds, leaves, and bark of this tree have traditionally been employed in anti-inflammatory, anti-hyperglycaemic, anti-diarrheal, and anti-ulcer medications (Badole and Bodhankar, 2010; Kesari et al., 2010a). The oil content of pongamia seed ranges from 35 to 40% of dry weight with its composition being oleic acid (55%), an ideal fatty acid for high-quality biodiesel (Sreeharsha et al., 2016). Apart from oleic acid, pongamia oil contains linoleic acid (18%), stearic acid (9%), and palmitic acid (8%) (Arote and Yeole, 2010). Protein and starch make up about 35 and 7% of the dry weight of seeds, respectively. Upon trans-esterification, the pongamia oil can be blended with diesel and utilized in automobiles without requiring any engine modifications. Pongamia seed oil differs from the other two promising biofuel plants, *Jatropha* of Euphorbiaceae and *Camelina* of Brassicaceae, in terms of oleic acid content and triacylglycerol (TAGs) composition (Mudalkar et al., 2014; Kumar et al., 2017). The tree can tolerate salt stress up to 300 mM through glycophytic and halophytic adaptive mechanisms in which Na^+^ sequestration in roots and K^+^ accumulation in leaves contribute to sustaining photosynthetic efficiency under salt stress (Marriboina et al., 2017).

Numerous studies have recently established elite pongamia accessions based on certain morphological, physiological, and agronomic traits, and a few studies have also documented genetic variability among pongamia accessions by using RAPD, ISSR, and AFLP markers (Kesari et al., 2010b; Kesari and Rangan, 2011; Rao et al., 2011). A variety of environmental factors influence the morphological and agronomic traits of wild pongamia accessions. Also, the capacity for carbon sequestration, photosynthetic activity, and yield patterns of pongamia accessions are unknown. As a result, there is a substantial need to analyze and document the level of carbon sequestration potential among naturally growing and widely distributed pongamia accessions to identify elite trees for biodiesel production. Long gestation periods, lengthy flowering cycles, flower drop, and inconsistent yields have become the bottlenecks for commercial pongamia cultivation. Furthermore, a lack of genomic resources limits crop improvement and breeding efforts, rendering wild pongamia accessions unreliable for consistent yields. Pongamia accessions that are currently accessible have adapted to a wide variety of edaphic and climatic conditions, indicating substantial genetic heterogeneity and low domestication. To maintain pure lines during long-term domestic cultivation for industrial uses, deeper characterization of pongamia elite accessions using current biotechnology, genetics, and genomics is required (Kesari et al., 2010b). This will also help to establish extensive plantations on marginal lands through *in vitro* culture techniques, which are otherwise impractical due to the inherent challenges in natural propagation. In this scenario, whole-genome sequencing is critical to understanding the structure, organization, and composition of the genome, which will give a substantial platform for designing crop improvement and molecular breeding initiatives.

Pongamia genome research has only recently gained momentum after its potential as a biofuel tree species was revealed. For the first time, Kazakoff et al. (2012) have disclosed the chloroplast and mitochondrial genome information of pongamia. Furthermore, using a flow cytometer, the 2C DNA content was measured to be 2.51 + 0.01 pg, with reference to *Z. mays* and *P. sativum* (Choudhury et al., 2014). Pongamia transcriptome and Expressed Sequence Tags (ESTs) gave important insights into lipid biosynthesis, photoperiod, and salt-responsive genes (Huang et al., 2016; Sreeharsha et al., 2016). This study was aimed at understanding the potential problems of pongamia, including its long gestation period, lengthy flowering cycles, susceptibility to gall midge disease, and other biotic factors through its genome. We report on the photosynthetic performance, biomass accumulation, and carbon sequestration potential of different pongamia accessions, as well as the quantity and quality of oil, which serve as crucial descriptors for elite pongamia accessions concerning commercial agronomic yields. Furthermore, the genome of a high-yielding, disease-free pongamia accession was sequenced and revealed the features of the genome to better understand its structure and organization. The major objective of the study is to report the sequence and structural information of genes involved in crucial metabolic pathways, including lipid metabolism, development, and flowering in pongamia, which play a significant role in further crop improvement programs of this potential biofuel tree species. To the best of our knowledge, this is the first study to reveal the genome sequence of a high-yielding pongamia, reporting 680 Mb of genome utilizing multiple Illumina sequencing platforms and hybrid assembly, followed by functional annotation.

## Materials and methods

### Plant material

**FIGURE 1:**
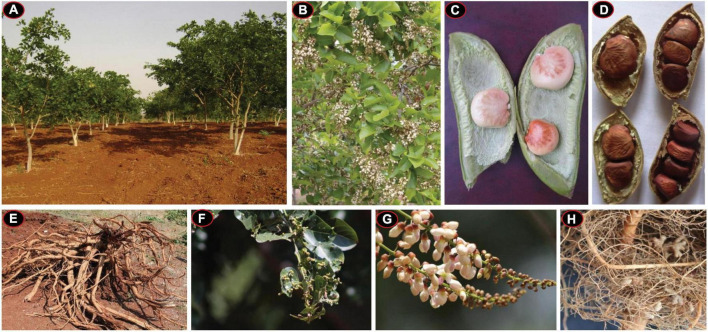
Morphological and phenological characteristics of a typical pongamia plant. **(A)** *Pongamia pinnata* plantation in an experimental farm. **(B)** Pongamia at a full-bloom stage. **(C)** An immature pod with a typical seed-arrangement pattern. **(D)** Mature pods bearing varied number of seeds. **(E)** Root biomass harvested. **(F)** Characteristic leaf buds of pongamia. **(G)** The Racemose type of inflorescence in pongamia. **(H)** Root nodules of pongamia.

Five different *Pongamia pinnata* accessions were collected from the nursery of Tree Oils India Limited (TOIL), Zaheerabad, Telangana state, India (latitude, 17*^o^*36′; longitude, 77*^o^*31′E; 622-m MSL) and raised in the experimental farm of University of Hyderabad, India, and grown with minimal agronomic inputs for 5 years. Each plant was planted in a 3-m-×-3-m spacing block planting model with initial application of organic fertilizer as a source of major and trace elements (Supplementary Figure 6). After attaining a reproductive phase, all the accessions were assessed for their morphological and physiological characteristics and yield potential. Typical morphological and phenological features of pongamia are depicted in Figure 1. For genome sequencing, leaves from high-yielding *P. pinnata* accession (TOIL055), which is a 5-year-old tree, were collected and snap frozen in aseptic conditions and stored at –80*^o^*C for further use.

### Photosynthetic leaf gas exchange measurements

Photosynthetic capacity of pongamia plants was assessed using leaf gas exchange parameters measured on fully expanded upper canopy leaves between 09:00 and 10:00 h, using a portable infrared CO_2_/H_2_O gas analyzer (IRGA) (LC Pro+, ADC BioScientific Ltd., United Kingdom) as described (Reddy et al., 2019). A saturating photosynthetically active radiation (PAR) of 1,600 μmol m^–2^ s^–1^ was supplied using a LED light source (LC pro Lamp 32070—Broad, ADC BioScientific Ltd., United Kingdom) attached to a leaf chamber. Air temperature was 25–26°C, and CO_2_ concentration was maintained at 390 μmol mol^–1^, and relative humidity was set to 55–60%. The net photosynthetic rate (P_*n*_), stomatal conductance (g_*s*_), the transpiration rate (E), and intercellular CO_2_ concentration (C_*i*_) were measured in representative plants. Leaf-level water use efficiency (WUE_*i*_) was calculated from gas exchange data by dividing the photosynthetic rate (P_*n*_) with the transpiration rate (E). The gas exchange measurements were recorded for a complete yield cycle, i.e., starting from April 2018 to March 2019 in periodic intervals of 1 month and represented as mean ± SD of ten replicative plants of each accession. ANOVA was performed to test the significance in difference between means of individual accessions.

### Growth, yield, and destructive biomass measurements

Morphological and phenological growth characteristics, including plant height, canopy width, and plant girth, were recorded for each accession after their gestation period. Reproductive yield data, including pod length, breadth, seed length, breadth, thickness, and 100-pod weight, 100-seed weight and total pod yield, were recorded for all the accessions from the date of flowering. Leaf length and breadth were measured using a portable laser leaf area meter (CI-202; CID Biosciences, Inc.) equipped with a laser scanner with a built-in control unit. Fresh and dry biomass of above and below ground tissues was determined after harvesting a 5-year-old tree. Carbon content in the stem was estimated by measuring the wood volume for each plant according to Newbould (1967) and specific gravity according to Negi et al. (2003). The amount of carbon dioxide sequestered in the above ground biomass was calculated as the weight of the carbon ×3.663 (Magnussen and Reed, 2004).

### Oil extraction and GC analysis

Oil was extracted from 5 pongamia accessions (pooled seeds of ten replicative plants of each accession) by using the Soxhlet extraction method using hexane as the solvent according to Singha et al. (2019). Briefly, seeds (5 g) were grounded to fine powder, and oil was extracted with 150 ml of hexane at distillation temperature for 3 h in the Soxhlet extractor using a heating mantle. Hexane was removed from the extracted oil using a rotary evaporator (Heidolph 514-01002-06-0, Germany) at 55*^o^*C under reduced pressure for 30 min. Later, the oil extracted from three technical replicates was used to prepare fatty acid methyl esters (FAMEs; biodiesel) using methanol: sulfuric acid (1:1 ratio) for GC analysis. Briefly, oil was dissolved in 1 ml of toluene and 5-ml methanol: sulfuric acid (2%). The whole mixture was heated at 60*^o^*C for 5 h. Later, 2 ml of potassium carbonate solution was added to the mixture, and then FAMEs were extracted with 5 ml of hexane: diethyl ether in the 1:1 ratio. The mixture was stirred vigorously, and the upper layer was collected and evaporated with N_2_ purging. The FAMEs were dissolved in 1-ml hexane for GC analysis. FAMEs were analyzed on an Agilent 6890N gas chromatograph with an Agilent 5975B inert XL mass-selective detector. Chromatographic separation was achieved using a DB-23 capillary column (J&W Scientific, Folsom CA; 30 m × 250 μm × 0.25 μm) with the following temperature program: initial temperature, 90*^o^*C, raised at 10*^o^*C/min to 165*^o^*C, held for 5 min, and then raised at 3*^o^*C/min to 230*^o^*C. The obtained peaks were compared with standard fatty acid mix (Supelco, Sigma Aldrich, United States), and the composition was quantified by measuring the peak area.

### DNA isolation, library preparation, and sequencing

Genomic DNA was isolated from leaf tissues of pongamia using a GenElute plant genomic DNA miniprep kit (Sigma-Aldrich, United States) according to the manufacturer’s instructions. To avoid the contamination with RNA and protein, the isolated DNA was subjected to RNaseA, proteinase K treatment. The genomic DNA was quantified by Qubit fluorometer (Thermo Fisher Scientific, United States) and used for library preparation. Multiple genomic DNA libraries for different sequencing platforms were generated for a pongamia DNA sample by following the Illumina library preparation protocol. Briefly, 1 μg of genomic DNA was fragmented using ultrasonicator (Covaris M220, United States) by following the manufacturer’s instructions, and the ends were repaired using SureSelect XT2 Library Prep Kit (Agilent, United States). The genomic DNA was cleaned up using AgencountAMPure XP SPRI beads (Beckman Coulter, United States). The DNA fragments were further adenylated at 3′ end and ligated with a paired-end adaptor. The indexed library was amplified, cleaned, and quantified by Qubit fluorometer using a DNA-HS kit (Thermo Fisher Scientific, United States). Two representative libraries from the same accession were loaded independently on different sequencing platforms, including HiSeq, MiSeq, and NextSeq. The generated reads were quality checked using a FastQC tool. Data were processed for adaptors and low-quality bases (the Phred score < Q30 was removed). Adaptors were clipped using the Cutadapt tools with >Q30 and minimum read length of 50 (Martin, 2011).

### *De novo* assembly, functional annotation

*De novo* assembly of genomic data was carried out with processed reads (generated from multiple libraries) to generate contigs using a Soap *de novo* assembler (Xie et al., 2014). Scaffolding over the contigs was done using a Redundans tool (Pryszcz and Gabaldón, 2016). The Redundans tool performs the scaffolding and gap closure using multiple libraries. To assess the read utilization, the genomic data generated were aligned back to the assembled genome using Bowtie2 (Langmead and Salzberg, 2012). For predicting genes, three approaches were followed, i.e., the RNA-seq method, the *de novo* method (using Augustus), and the homology-based method. Homology-based gene prediction using *Cajanus cajan*, *Arabidopsis thaliana, Medicago truncatula*, and *Glycine max* gene models and *de novo*-based gene prediction using Augustus were carried out. Gene models generated using the *de novo*-based method (Augustus) were utilized for further downstream annotation. The predicted proteins were similarity searched against the UniProt Viridiplantae (6,386,419) protein database using the DIAMOND9 BLASTP program with an e-value cut-off of 10^–5^ to assign GO (Gene Ontology) terms and annotated into a biological process, molecular function, and a cellular component. Alignment of transcriptomic reads was carried out using a hisat2 (splice aligner) tool, and then a cufflinks tool was used to assemble the genes (Trapnell et al., 2012; Kim et al., 2015).

### Repetitive sequences and organellar genome analysis

Two approaches were followed to identify repetitive DNA in pongamia. *De novo* repeat identification was done using RepeatModeler while homology analysis against the RepBase library was carried out using RepeatMasker (Chen, 2004). The pongamia mitochondrial genome was mined and compared with *Glycine max* using a BLAST Ring Image Generator (BRIG) tool (Alikhan et al., 2011).

### Phylogeny tree construction, genome comparison, and simple-sequence-repeat marker identification

For phylogenetic tree construction, protein sequences of *Glycine max*, *Medicago truncatula, Eucalyptus grandis, Jatropha curcas, Populus trichocarpa*, and *Cajanus cajan* were retrieved from NCBI. CD-hit was executed to obtain orthologous clusters with 90% alignment coverage (Li and Godzik, 2006). Multiple sequence alignment was performed on generated clusters using ClustalW, and, subsequently, the Gblocks program was used to extract the conserved blocks from the alignment (Larkin et al., 2007). On the basis of concatenated blocks, the phylogenetic tree was constructed using the tool “Seaview,” where the parsimony method with 1,000 bootstrap replicates was used (Gouy et al., 2009). A Benchmarking Universal Single-Copy Orthologs (BUSCO) analysis was performed using the ortholog clusters from OrthoDB as well as a BUSCO plant gene set to find the completeness of the genome assembly (Simão et al., 2015; Zdobnov et al., 2017)^1^. For SSR marker identification, repeats were identified in each scaffold sequence using MISA11 (the MicroSAtellite identification tool) (Beier et al., 2017).

### Pathway identification

The predicted proteins were uploaded in the KEGG-KAAS10 server for pathway identification using *Glycine max*, *Medicago truncatula*, *Lotus japonicus*, *Arachis duranensis*, and *Arachis ipaensis* as reference organisms to identify the enriched metabolic pathways in various gene sets (Moriya et al., 2007).

## Results

### Photosynthetic physiology

**FIGURE 2:**
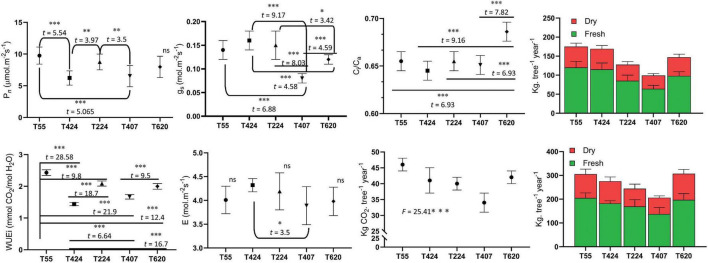
Leaf gas exchange parameters of different accessions of pongamia along with above and belowground tissues biomass and amount of CO_2_ sequestered per plant. Data are the mean ± SD. The difference between variables of individual accessions was indicated with *t* and *F* statistic at *p = 0.1, ^**^p = 0.05, and ^***^p = 0.01 level of significance.

Photosynthetic leaf gas exchange characteristics exhibited significant variation among the 5 accessions (Figure 2). TOIL055 showed a maximum photosynthetic rate (P*_*n*_*) of 9.76 μmol.m^–2^s^–1^, which was significantly higher than the rest of the accessions (*F* = 10.89; *p* < 0.0001) (Figure 2). There was no significant difference in P*_*n*_* values of TOIL424 and TOIL407, which showed assimilation rates of 6.23 and 6.53 μmol.m^–2^s^–1^, respectively (Figure 2). Similarly, stomatal conductance (g*_*s*_*) (*F* = 26.32; *p* < 0.0001), the transpiration rate (E) (*F* = 2.98; *p* < 0.028), and leaf water use efficiency (WUEi) (*F* = 245.1; *p* < 0.0001) were significantly higher in TOIL055 when compared to other accessions (Figure 2). Internal CO_2_ concentration (C_*i*_) in the leaf also showed significant difference among the accessions (*F* = 25.48; *p* < 0.0001) (Figure 2).

### Growth, yield, and destructive biomass measurements

Morphological growth characteristics like plant height, canopy width, plant girth and leaf area were measured for three representative plants of each accession and documented as mean ± SD with significance values in their variance (Table 1). The plant height and canopy width ranged from 4.5 (TOIL620) to 6.5 (TOIL224) and 6.1 (TOIL620) to 7.5 m (TOIL224), respectively, among the accessions (Table 1). TOIL224 showed maximum plant girth (80 cm), while TOIL407 showed minimum plant girth (71 cm) (Table 1). Leaf length and leaf width have not shown any significant difference among the five accessions (Table 1). In addition, pod and seed characteristics were measured from replicative plants after pod harvesting (Table 1). A single pod of pongamia plant can bear 1–4 seeds per pod, depending upon the accession (Figure 1D). The characteristics, including pod length (*F* = 98.1; *p* < 0.0001), pod breadth (*F* = 61.28; *p* < 0.0001), seed length (*F* = 29.89; *p* < 0.0001), breadth (*F* = 21.56; *p* < 0.0001), and thickness (*F* = 18.9; *p* < 0.0001), differ significantly among the five accessions (Table 1). Although 100-pod weight was not significantly differed between accessions, the values of 100-seed weight were significantly high in TOIL055 than in other accessions (*F* = 9.43; *p* < 0.0001) (Table 1). Reproductive yield was recorded for each accession and reported as mean ± SD (Table 1). Among all the elite trees, the highest pod yield of the 27-kg tree^–1^ year^–1^ was recorded in TOIL55, while the lowest-yield 20.5 kg tree^–1^ year^–1^ was recorded in TOIL620 (Table 1). The data of 14 morphological and phenological parameters of all elite trees were checked for any interrelation using the Pearson Correlation Coefficient, and the *r*^2^ values were tested for significant difference and represented in Supplementary Table 1. Plant height scaled positively with canopy width, seed length, 100-seed weight, and pod yield (Supplementary Table 1). Canopy width and leaf width also scaled positively with 100-seed weight and pod yield (Supplementary Table 1). Leaf characteristics like leaf length and leaf width were associated with pod yield, but the same were not correlated with 100- seed weight. Among yield parameters, 100-seed weight and pod yield were positively correlated with seed thickness and 100-pod weight, respectively (Supplementary Table 1). Interestingly, the oil content of seeds was not correlated with either of seed width or seed thickness. Furthermore, pod yield and seed weight had no relation to the oil content of seeds.

**TABLE 1:** Morphological, phenological, and reproductive yield characteristics of pongamia.

	TOL055	TOL424	TOL224	TOL407	TOL620	Variance (F; *p*-value)
Plant height (m)	5.40 ± 0.20	5.87 ± 0.21	6.50 ± 0.16	5.38 ± 0.39	4.50 ± 0.22	86.91***
Canopy width (cm)	7.00 ± 1.02	6.95 ± 0.98	7.55 ± 0.92	6.85 ± 1.10	6.10 ± 1.04	2.62*
Plant girth (cm)	72.00 ± 2.10	78.00 ± 2.34	80.00 ± 1.98	71.00 ± 1.11	72.00 ± 3.45	31.18***
Leaf length (cm)	9.62 ± 0.81	8.91 ± 0.22	7.77 ± 0.12	7.63 ± 0.55	8.15 ± 0.81	22.15***
Leaf width (cm)	6.36 ± 0.12	6.14 ± 0.21	5.17 ± 0.23	5.65 ± 0.11	4.92 ± 0.23	112.5***
Pod length (mm)	42.00 ± 1.21	45.00 ± 0.98	40.00 ± 1.21	42.00 ± 1.01	52.00 ± 0.98	189.1***
Pod breadth (mm)	20.00 ± 0.89	21.00 ± 0.12	20.00 ± 0.98	22.00 ± 0.43	26.00 ± 0.76	122.6***
100-pod weight (kg)	2.51 ± 0.58	2.72 ± 0.66	2.55 ± 0.85	2.82 ± 0.92	3.23 ± 0.76	ns
Seed length (mm)	22.32 ± 1.01	24.63 ± 0.87	24.61 ± 0.98	22.24 ± 1.01	20.82 ± 0.89	29.89***
Seed breadth (mm)	12.48 ± 0.76	13.67 ± 1.01	14.23 ± 0.99	14.28 ± 0.32	15.95 ± 0.98	21.56***
Seed thickness (mm)	7.59 ± 0.66	8.12 ± 0.85	8.72 ± 0.92	6.93 ± 0.76	6.05 ± 0.5	18.9***
100-seed weight (g)	168.16 ± 12.3	139.76 ± 22.2	138.19 ± 10.9	128.92 ± 13.4	140.82 ± 14.5	9.43***
Pod yield (KG)	27.08 ± 1.54	22.74 ± 1.91	21.93 ± 1.78	20.92 ± 1.53	20.53 ± 1.89	22.88***
Oil content (%)	35.25 ± 0.91	33.98 ± 0.45	28.63 ± 1.34	31.49 ± 1.02	31.38 ± 2.78	28.4***

Values were represented as mean ± SD. The difference in means was indicated by F statistic at 0.1 (*), 0.05 (**), and 0.01 (***) level of significance.

The pongamia tree was completely uprooted to measure biomass of aboveground and belowground tissues independently (Figure 1E). Destructive biomass measurements clearly demonstrated that TOIL055 accession accumulated a 205 kg tree^–1^ total aboveground fresh biomass and a 120 kg tree^–1^ total belowground fresh biomass, whereas above and belowground dry biomass of same accession was 100 and 55 kg trees^–1^, respectively (Figure 2). Remaining accessions of pongamia have shown lower fresh and dry biomass after 5 years of growth (Figure 2). The carbon sequestration potential of pongamia was estimated by calculating the amount of CO_2_ sequestered per year. There was significant difference among pongamia accessions in CO_2_ sequestration capacity wherein TOIL055 accession showed 46 kg of CO_2_ per tree per year, which was higher when compared to the other accessions (*F* = 25.4; *p* = 0.0001) (Figure 2).

### Oil content and GC/MS analysis

Oil content measured in different accessions exhibited significant variation among the elite trees (Table 1). The oil content was highest (36%) in TOIL055 seeds, which is a high-yielding variety, whereas seeds obtained from TOIL224 showed the lowest oil content (28.6%) (*F* = 28.4; *p* = 0.0001). GC/MS analysis of FAMEs prepared from seed oil revealed the quality of oil in various elite varieties (Table 2). The quality of seed oil has shown no variation among the accessions. The fatty acid composition of seed oil was predominated by oleic acid followed by linoleic acid, palmitic acid, stearic acid, along with linolenic acid and behenic acid, in most of the accessions (Table 2).

**TABLE 2:** Fatty acid methyl esters (FAMEs) analysis of oil extracted from five different pongamia accessions.

Fatty acid methyl ester (%)	TOIL055	TOIL424	TOIL224	TOIL407	TOIL620
Palmitic acid (16:0)	9.7	8.4	9.2	9.9	7.9
Stearic acid (18:0)	7.9	8.1	5.7	8.4	6.9
Oleic acid (18:1)	55.2	52.9	53.5	50.8	58.1
Linoleic acid (18:2)	14.1	16.8	15.6	14.8	13.7
Linolenic acid (18:3)	3.6	2.7	4.2	3.9	4.8
Behenic acid (22:0)	3.4	3.2	2.8	4.7	3.7
Others	6.1	7.9	9.0	7.5	4.9

Individual FAMEs were indicated as the peak area percentage of the total area obtained.

### Genome sequencing, assembly, and validation

The draft genome of *P. pinnata* was assembled by paired-end reads, generated by sequencing three hybrid libraries with different read lengths. One library each of Illumina HiSeq4000, NextSeq500, and MiSeq, with 150, 150, and 300 read lengths, respectively, were constructed. The sequencing resulted in generation of ∼408 million paired end reads, which were further processed to generate 356 million high-quality reads (Supplementary Table 2). These reads were subsequently assembled to generate 204,148 contigs, and these contigs were further merged into 199,825 scaffolds by closing the gaps. The longest scaffold had a sequence length of 48.3 Kb and a minimum sequence length of 960, with an average length of 3,432 bp. The statistics of contigs and scaffolds, including average sequence length, N50 value, number of non-ATGC characters, and number of sequences of various sizes, are given in Table 3. Complete single-copy and duplicated BUSCOs correspond to 71% of 5,366 searched BUSCO groups, while there were 26.3% missed BUSCOs obtained from the sequence alignment (Supplementary Table 3). The genomic data generated were aligned back to the assembled genome to find the read utilization percentage, which was found to be 80.63, 83.26, and 80.32% for HiSeq, NextSeq, and MiSeq libraries, respectively. With the help of the three Illumina short reads library data, the assembly showed relatively good N50 and BUSCOs. In order to validate the genome assembly, we aligned 22 million reads generated using the EST library from our previous study. Nearly 60% of reads were aligned with the genome, and 24,695 transcripts were mapped to the respective scaffolds with greater than 95% identity (Supplementary Data 1).

**TABLE 3:** Pongamia genome assembly statistics showing the contigs and scaffold features.

Parameter	Contigs	Scaffolds
Number of Sequences	204148	199825
Total Sequences Length	685500274	685887678
Maximum Sequence Length	45815	48003
Minimum Sequence Length	960	960
Average Sequence Length	3357.86	3432.44
Number of non-ATGC Characters	260696	151517
% of non-ATGC Characters	0.038	0.0221
Sequences ≥ 100 bp	204148	199825
Sequences ≥ 200 bp	204148	199825
Sequences ≥ 500 bp	204148	199825
Sequences ≥ 1 Kbp	204142	199821
Sequences ≥ 10 Kbp	9013	9473
N50 Value	4599	4752

### Gene prediction and functional annotation

**FIGURE 3:**
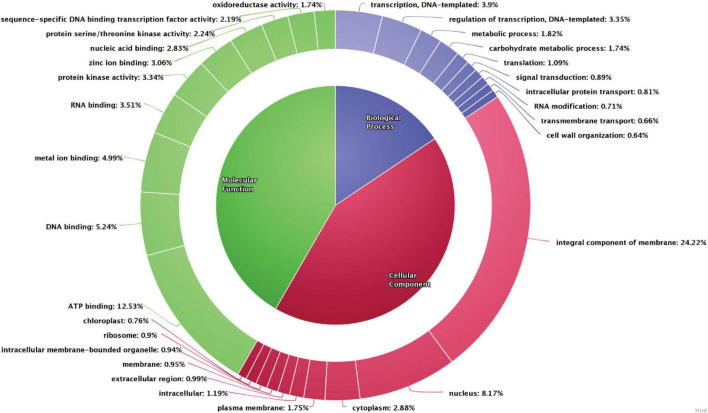
Gene ontology distribution of the scaffolds into the biological process, molecular function, and the cellular component. The number of scaffolds encoded for each category is represented.

The scaffolds were used to predict gene models using *de novo*, RNASeq, and homology-based methods, which resulted in identification of 26,633 genes. Gene models identified using the *de novo* method were further utilized for functional annotation. Functional annotation classifies the data into various categories, such as the biological process, cellular components, and molecular functions wherein each scaffold is assigned to a particular category based on the sequence similarity with the existing databases. In the pongamia genome, around 82.9% of predicted genes were annotated against 30,000 proteins from the existing protein databases (UniProt, TrEmbL, and Swiss-Prot), and another 6,162 unannotated proteins were also identified (Supplementary Data 2). These proteins belonged to various categories, such as the biological process, the cellular component, and the molecular function (Figure 3). A large number of scaffolds were distributed in the cellular processes category (55.28%), of which the integral component of membrane, ATP binding, nucleus, and cytoplasm represented the maximum number of scaffolds (24.22, 12.53, 8.17, and 2.88%, respectively), followed by plasma membrane (1.75%), intracellular proteins (1.19%), extracellular region proteins (0.99%), membrane proteins (0.95%), intracellular membrane-bound organelle (0.94%), ribosome (0.9%), and chloroplast (0.76%) (Figure 3). The second largest component of Gene Ontology was molecular function, with a percentage distribution of 29.14, among which 5.24% of scaffolds represented DNA-binding proteins, followed by metal ion-binding proteins (4.99%), RNA binding (3.51%), protein kinase activity (3.34%), zinc ion binding (3.06%), nucleic acid binding (2.83%), protein serine/threonine kinase activity (2.24%), proteins with sequence-specific DNA binding, transcription factor activity (2.19%), and oxidoreductase activity (1.74%). Scaffolds distributed in the biological process category were majorly represented by proteins involved in transcription, regulation of transcription, the metabolic process, and the carbohydrate metabolic process (3.9, 3.35, 1.82, and 1.74%, respectively), followed by translation (1.09%), signal transduction (0.89%), intracellular protein transport (0.81%), RNA modification (0.71%), transmembrane transport (0.66%), and cell wall organization (0.64%). Gene ontology studies covered proteins belonging to all major categories, thus representing the depth of analysis.

### Genome elements and structural features

In our study, a total of 15.74% of the base pairs were aligned with repetitive sequences with a reference-based repeat masker wherein 10.78% were retroelements, including, SINE’s, Penelope, and LINE’s (Table 4). The unified classification system grouped transposable elements systematically into 2 major classes, 9 orders, and 29 superfamilies based on the mode of chromosomal integration, gene structure, and sequence similarity. In pongamia, the major categories in retroelements are RTE/Bov-B (0.09%), L1/CIN4 (0.61%), LTR elements (10.06%), Ty1/Copia (4.87), and Gypsy/DIRS1 (4.9%). Whereas, the genome also represented 14.1% of DNA

**TABLE 4:** *De novo* and reference-based prediction of repetitive sequences in the pongamia genome.

*De novo* repeat prediction			
**Transposable element**	**Number of elements**	**Length occupied (bp)**	**Sequence (%)**
LINEs	9510	3289383	0.48
LINE1	8680	3190382	0.47
LTR elements	71293	47587384	6.94
DNA elements	9664	4379873	0.64
Unclassified	769940	232655702	33.92
Total interspersed repeats		287912342	41.98
Simple repeats	23661	10943688	1.6
Low complexity	1829	105929	0.02
**Reference based repeat prediction**			
Retroelements	145171	73924657	10.78
SINEs1	466	52333	0.01
Penelope	1	45	0.00
LINEs	12621	4844989	0.71
R2/R4/NeSL	3	135	0.00
RTE/Bov-B	2514	635344	0.09
L1/CIN4	10071	4206842	0.61
LTR elements	132084	69027335	10.06
Ty1/Copia	51644	33385530	4.87
Gypsy/DIRS1	74772	34094727	4.97
DNA transposons	47091	9698372	1.41
hobo-Activator	14111	3386899	0.49
Tc1-IS630-Pogo	838	85955	0.01
Tourist/Harbinger	4735	1120082	0.16
Unclassified	6062	1778247	0.26
Total interspersed repeats	698769	85401276	12.45
Small RNA	208	34628	0.01
Satellites	227	21195	0.0
Simple repeats	356234	16659552	2.43
Low complexity	104701	5949655	0.87

transposons, 0.49% hobo-activator, 0.01% Tc1-IS630-Pogo, 0.16% of Tourist/Harbinger, Interspersed repeats (12.45%), Small RNA’s (0.01%), Simple repeats (2.43%) and low complexity repeats (0.87%) were also identified in the pongamia genome (Table 4). *De novo* repeat prediction of the pongamia genome by a repeat modeler gave 43.6% of the bases as repetitive sequences of which 0.48% were LINEs, 6.94% were LTR elements, 0.64% DNA elements, 42% of total interspersed repeats, 1.6% of simple repeats, and 0.02% of low-complexity elements along with 33.9% of unclassified repeats (Table 4).

We examined a total of 199,825 sequences and identified 406,385 SSRs in which mono-nucleotide SSRs represented the largest fraction (53.7%), followed by tetra-nucleotide (21%), and di-nucleotide SSRs (17.5%) (Supplementary Table 4). Out of the total examined sequences, 143,942 sequences had at least 1 SSR, while 100,447 sequences had more than 1 SSR. Pongamia scaffolds also contained a quite significant number of tri—(7.3%), penta—(0.3%), and hexa nucleotide (0.15%) SSRs, although their representation is small in the total SSR pool (Supplementary Table 3).

**FIGURE 4:**
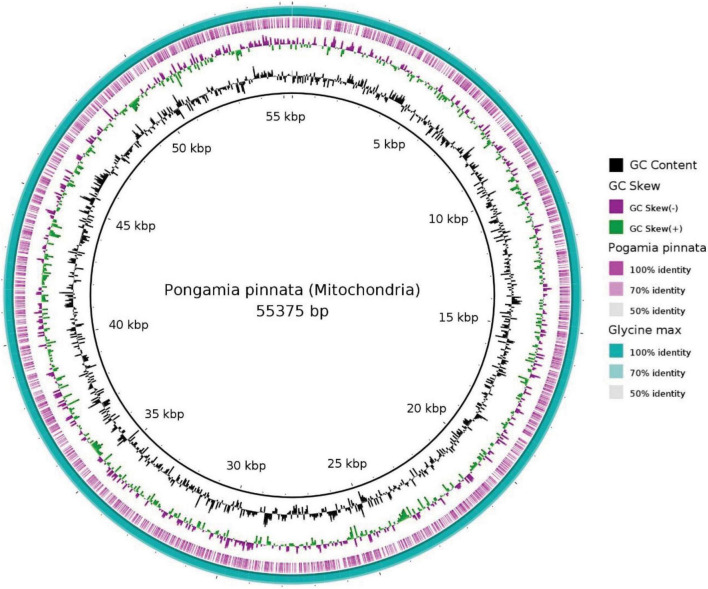
Mitochondrial genome architecture of *Pongamia pinnata* with comparison to *Glycine max*. Moving from outside to inside, the first circle shows the blast alignment of the *Glycine max*, while the second purple ring represents blast alignment of the *Pongamia pinnata*. The third circle shows the GC Skew with above-zero values in green and below-zero values in purple. The fourth circle shows the mean centered GC content, with the average GC as the baseline and outward projections as higher than average and inwardly projections as lower than average. The inner circle shows the position coordinates for the mitochondrial genome sequence.

In the current study, 55.3 Kb of the pongamia mitochondrial genome was compared with that of *G. max* and identified 81 scaffolds, representing different genes of mitochondrial metabolism (Supplementary Data 3). Figure 4 shows the BRIG image for blastn comparisons and reveals that the pongamia and soybean mitochondrial assemblies have a nearly complete genome identity. We mined sequences for ATPase subunits, cytochrome c biogenesis proteins, cytochrome c oxidase subunits, maturase, NADH dehydrogenase subunits, ribosomal proteins, and transport membrane proteins (Supplementary Data 3).

### Genome comparison and phylogenetic analysis

**FIGURE 5:**
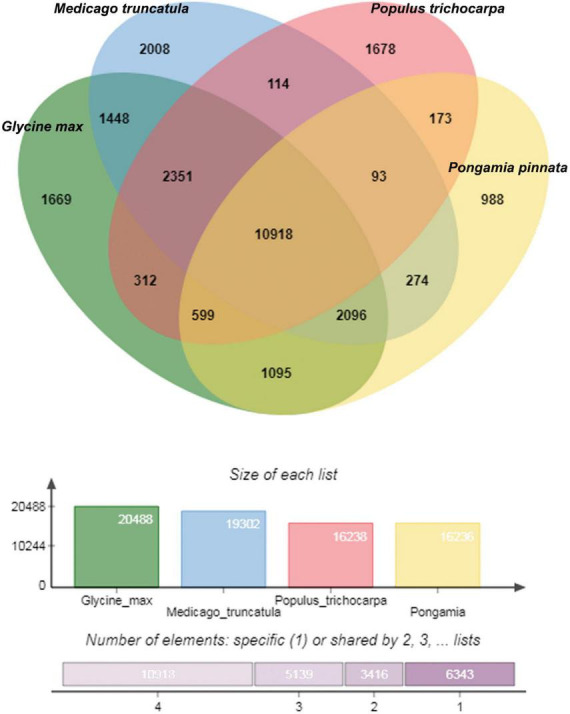
A Venn diagram showing the uniformity between the orthologous proteins among four different plant species. A total of 16,179 proteins of pongamia identified in the current study were compared with that of *Medicago truncatula, Populus trichocarpa*, and *Glycine max* and found out the commonly shared proteins between 2, 3, and 4 species. Also represented 1,002 unique or uncharacterized proteins, which were not matched with any other protein in the existing databases.

**FIGURE 6:**
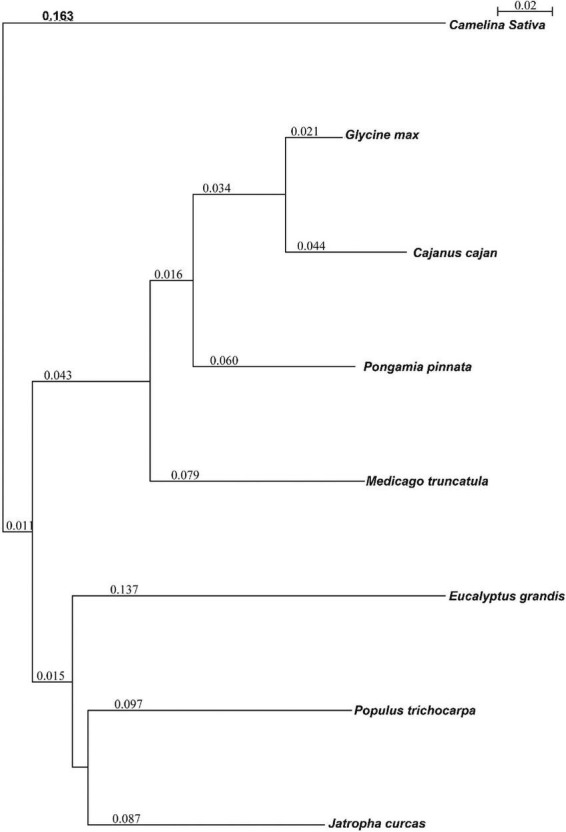
The phylogenetic relationship of pongamia with other related organisms based on the orthologous proteins. A Seaview program was used to compute the phylogenetic tree. The program used a fast distance-based (Neighbor-Joining) method, BioNJ, to compute a full initial tree. The values present in the image represent the branch length.

Orthological studies were conducted to see the similarity of pongamia with that of other model legume plants and tree species by gathering the protein sequences from multiple sources, including TrEmbL, UniProt, and EST databases. Nearly, 16,236 functional proteins from *P. pinnata* identified in the current study were compared with that of 20,488 orthologous proteins of *Glycine max*, 19,302 of *Medicago truncatula*, and 16,238 of *Populus trichocarpa* (Figure 5). Among the analyzed proteins, 10,918 were common in all the four plant species, whereas 6,117 and 3,902 were common among 3 and 2 plant species, respectively, while 988 proteins annotated in this study were exclusively found in pongamia, showing the specificity of the proteins with only pongamia (Figure 5). Phylogenetic analysis was performed to know the evolutionary conservation of pongamia, revealing a monophyletic clade of Fabaceae members, which were separated from other tree species (Figure 6). In total, 65,536 orthologous clusters were extracted from whole genomes of different plant species and identified 25 single-copy orthologous clusters among diversified plant species based on which the phylogeny was deduced (Supplementary Data 4). Interestingly, pongamia out-grouped with pigeonpea and soybean, which are leguminous crops, while it converged with jatropha and populus that are biofuel yielding and slow-growing tree species, respectively. We also compared the phylogenetic relationship of pongamia with other plant species with respect to complete lipid metabolism genes (Supplementary Figure 2A). Similarly, based on the phylogeny of environmental interaction genes, pongamia was placed in between *Cajanus cajan* and *Medicago truncatula*, showing maximum homology with these legumes (Supplementary Figure 2B). We found that the photosynthesis-related genes were conserved mostly among Fabaceae members, forming a monophyletic group (Supplementary Figure 2C). Furthermore, the phylogeny of transcription factor genes revealed similar relationship as that of other metabolic pathway genes, showing maximum homology with legume members (Supplementary Figure 2D).

### Characteristic features of protein coding genes

Ortholog assignment and mapping of the contigs to the biological pathways were performed using KEGG automatic annotation server (KAAS) (Supplementary Data 5). All the contigs were compared against the KEGG database using BLASTX with a threshold bi-score of 60 (default). It assigned Enzyme Commission numbers for 12,581 scaffolds, and they were mapped to respective categories (Supplementary Figure 1). Among the mapped scaffolds, 3,115 were identified as genes involved in metabolic pathways of major biomolecules, such as carbohydrates (857), amino acids (495), lipids (416), nucleotides (216), cofactors (226), glycans (102), and terpenoids (152), while 3,653 scaffolds representing 1,954 different unigenes were belonged to genetic information and processing, performing various functions, including transcription, translation, replication, and repair, as well as folding, sorting, and degradation. Another major KEGG group was signaling and cellular processes wherein 1,013 scaffolds encode 435 unigenes that perform various roles in endocytosis, phagocytosis, transport, and catabolism. Among these, 69 unique genes that belong to peroxisome metabolism were identified. Our data also identified genes involved in major signaling mechanisms, such as the MAPK- signaling pathway (115), the phosphotidyl inositol signaling system (56), as well as plant hormone signal transduction (217). Around 5% of the functional proteins were either uncharacterized or unannotated with the KAAS database (Supplementary Figure 1), while environmental adaptation (2%) represented a minor portion among different categories (Supplementary Figure 1). The genes involved in the following important metabolic pathways were studied further because of their significant interest to the improvement of pongamia.

#### Lipid metabolism

In the draft genome of pongamia, 311 scaffolds represented the genes involved in lipid metabolism, which were further categorized into fatty acid biosynthesis (22), elongation (24), degradation (26), ketone body synthesis and degradation (5), cutin, suberin, and wax biosynthesis (20), steroid biosynthesis (24), glycerolipid metabolism (47), glycerophospho lipid metabolism (49), ether lipid metabolism, arachidonic acid metabolism, and sphingolipid metabolism (Supplementary Data 6). The crucial gene features, including gene length, number and size of the exons, and introns of enzymes involved at various stages of fatty acid biosynthesis, are given in Table 3. The sequence information of genes involved in priming of acetyl CoA and malonyl CoA into long-chain acyl A and subsequent generation of free FAs by thioesterases and long-chain acyl-CoA synthetases to final production of TAGs were deduced in this study. The largest gene with 14,548 bp, possessing 33 exons and 32 intron regions corresponds to ACCase biotin carboxylase (BC), which is one of the subunits of the multi-subunit acetyl-CoA carboxylase (ACCase) complex that limits the oil accumulation in the seeds, while long-chain acyl Co-A Synthetase represented the longest intron of 9754 bp (Table 5). The fatty acid metabolism genes have majorly shown homology with *Glycine max*, indicating the conserved nature of lipid biosynthesis along the legume family members. The enzymes involved in unsaturated fatty acid biosynthesis, including SAD, FAD2, FAD8 that participate in biosynthesis of oleic acid, linoleic acid, and linolenic acid, respectively, showed maximum homology with legume members other than *Glycine max* (Table 5). The phylogenetic analysis of important lipid biosynthetic enzymes revealed the common ancestors for their evolutionary divergence. PpFAD2, PpFAD3, and PpFAD6 formed a distinct monophyletic group with *Glycine max* and *Medicago truncatula*, which belong to the same family (Supplementary Figure 3). However, PpFAD3 showed some sequence dissimilarity, thus forming a separate clade in the Fabaceae monophyletic group (Supplementary Figure 3).

**TABLE 5:** Gene characteristics of key enzymes involved in lipid metabolism of pongamia.

Name	Symbol	Homology (%)	Gene length (bp)	No. of exons	No. of introns	Total exon size	Total intron size	Longest exon (bp)	Longest intron (bp)
**Acetyl CoA biosynthesis**									
PDHC-E1 Component α subunit	PDHC	*Glycine max* (90.7)	2495	3	2	1956	535	1034	452
ATP-Citrate lyase	CL	*Glycine soja* (97.3)	2794	10	9	1545	1231	473	225
Acetyl-CoA synthetase	ACSS	*Cajanus cajan* (88.6)	6812	16	15	1785	4997	320	957
Acetyl-CoA acetyl transferase	ACT	*Glycine soja* (85.3)	5992	11	09	1466	2547	358	896
**Fatty acid biosynthesis**									
ACCase carboxyl transferase α	accA	*Glycine soja* (78.7)	6749	12	9	2801	2963	1053	1514
ACCase carboxyl transferase β	accB	*Glycine max* (90)	3527	7	6	1376	2139	375	1231
ACCase biotin carboxylase	ACACA	*Cicer arietinum* (74.8)	14548	33	32	7163	7321	2153	1491
ACCase homomeric protein	ACAC	*Glycine soja* (84.3)	5078	11	9	1466	2547	358	896
Fatty acyl-ACP thioesterase B	FATB	*Cajanus cajan* (94.6)	4554	7	5	1731	2245	518	805
Fatty acyl-ACP thioesterase A	FATA	*Glycine soja* (82.4)	5724	8	7	1965	3745	739	1208
Long-chain acyl-CoA synthetase	ACSL	*Morus notabilis* (74.1)	11671	15	14	1889	9754	251	2447
**Fatty acid elongation**									
Ketoacyl-CoA synthase	KCS	*Glycine soja* (94)	4824	3	2	1883	2937	763	1564
Very-long-chain enoyl-CoA reductase	ECR	*Medicago truncatula* (79)	3707	9	8	1404	2287	492	808
VLC hydroxyacyl-CoA dehydratase	PHS1	*Glycine max* (89)	3875	9	8	1093	2766	377	1081
Very-long-chain 3-oxoacyl-CoA reductase	HSDB	*Glycine soja* (86)	2491	2	1	807	1682	457	1682
**Biosynthesis of unsaturated fatty acids**									
Acyl-ACP desaturase	FAB2	*Trifolium pratense* (89.6)	3370	2	1	1810	1558	1130	1558
ω 6 fatty acid desaturase (Δ 12 desaturase)	FAD2	*Vigna radiata* (90.6)	3071	3	1	1436	590	698	590
ω 3 fatty acid desaturase (Δ 15 desaturase)	FAD3	*Cajanus cajan* (77.6)	2989	8	7	2086	889	1038	300
**TAG biosynthesis**									
Glycerol-3-phosphate acyltransferase	GPAT	*Glycine max* (78.9)	2702	2	1	2014	686	1265	686
Diacylglycerol O-acyltransferase 1	DGAT1	*Glycine soja* (73.9)	5571	12	11	2467	3082	999	664
Diacylglycerol O-acyltransferase 2	DGAT2		5342	6	5	1791	3541	1105	1571
Diacylglycerol O-acyltransferase 3	DGAT3	*Glycine soja* (89)	1805	2	1	1480	323	1008	323
LPA O-acyltransferase	LPAT		3515	9	8	1897	1602	408	485
Lysophosphatidylcholine acyltransferase	LPCAT	*Cajanus cajan* (86.8)	9351	14	13	2105	7220	719	1401
Phospholipid: diacylglycerol acyltransferase	PDAT	*Cajanus cajan* (83.2)	5100	8	7	3273	1813	1367	742
**Fatty acid degradation**									
Alcohol dehydrogenase	ADH	*Quercus suber* (82.4)	1505	4	3	778	721	420	429
Acyl-CoA oxidase	AOX	*Glycine max* (94.2)	7432	14	13	2490	4916	448	1022
S-glutathione dehydrogenase	GDH	*Cicer arietinum* (87)	4232	10	9	1344	2870	325	702
Aldehyde dehydrogenase	ALDH	*Cajanus cajan* (97.1)	5512	10	9	1970	3524	434	1203
3-hydroxyacyl-CoA dehydrogenase	MFP2	*Vigna radiata* (88.3)	6785	18	17	2282	4469	302	1059
Acyl-CoA dehydrogenase	ACADM	*Medicago truncatula* (83)	1221	3	2	742	475	474	388
Phospholipase A1	DAD1	*Cicer arietinum* (7.09)	1673	1	0	1673	0	1673	NA
TAG lipase/phospholipase A2	TGL4	*Cajanus cajan* (87)	4643	3	2	2879	1760	1529	1484
Phospholipase C	PLC	*Cajanus cajan* (90)	3486	4	3	2446	1034	1633	486

#### Circadian rhythms, flowering, and plant pathogen interaction

We successfully identified 35 genes encoding various proteins that are involved in circadian rhythms and vernalization pathways of pongamia (Supplementary Data 7). The important genes among these are phytochrome-interacting factors (PIFs), EARLY FLOWERING 3, GIGANTEA, cryptochromes, phytochromes, chalcone synthase, Pseudo-response regulator 1, 5, and 7, Flowering locus T, as well as key transcription factors, including HY5, MYB-related transcription factor LHY, and TCP21 (Table 6). In Arabidopsis and other model plants, the GIGANTEA-CONSTANS-FLOWERING LOCUS T-APT signaling cascade acts as a major flowering regulatory mechanism (Roux et al., 2006). The size of the largest gene was 6,247 bp, having 4 exons and 3 introns coding for phytochrome B. The same gene had also represented the longest exon of 2,086 bp, while the longest intron was found in ELF3 (Table 6). All the flowering-related genes have shown maximum homology with legume family members wherein the majority of genes have shown similarity with *G. max* (Table 6). The sequence analysis of key flowering genes, including flowering locus T, gigantean, and PRR1, had shown their phylogenetic relationship with other legumes and biofuel plants (Supplementary Figure 4). Similarly, there are 122 genes regulating plant–pathogen interaction in pongamia (Supplementary Data 7). Calcium-binding proteins, signaling components, transcription factors, molecular chaperons, and gated ion channels are among the identified genes related to plant-pathogen signaling in pongamia (Supplementary Data 7).

**TABLE 6:** Gene characteristics of key enzymes involved in circadian rhythms and flowering regulation in pongamia.

Name	Symbol	Homology (%)	Gene length (bp)	No. of exons	No. of introns	Total exon size	Total intron size	Longest exon (bp)	Longest intron (bp)
Pseudo-response regulator 1	PRR1	*Glycine max* (82)	4534	7	6	1873	2649	1123	928
Pseudo-response regulator 7	PRR7	*Glycine max* (86)	2407	3	2	1683	720	804	632
Pseudo-response regulator 5	PRR5	*Glycine max* (79)	5743	8	7	2987	2742	893	1216
Cryptochrome 1	CRY1	*Phaseolus vulgaris* (88)	4346	5	3	2354	1720	903	611
Cryptochrome 2	CRY2	*Cajanus cajan* (92)	4671	6	5	2487	2174	706	622
EARLY FLOWERING 3	ELF3	*Cajanus cajan* (72)	5244	5	3	3037	2000	1425	1658
Phytochrome A	PHYA	*Cajanus cajan* (90)	6127	5	3	4120	1336	2078	615
Phytochrome B	PHYB	*Glycine max* (92)	6247	4	3	4155	2086	2266	797
Phytochrome-interacting factor 3	PIF3	*Glycine soja* (79)	5089	11	9	3038	1816	1254	666
Clock-associated PAS protein ZTL	ZTL	*Vigna radiata* (97)	2648	2	1	2468	178	2394	178
Phytochrome-interacting factor 3	PIF3	*Phaseolus vulgaris* (56)	2566	6	5	2008	548	954	121
Chalcone synthase	CHS	*Glycine soja* (96)	1915	2	1	1426	487	1155	487
FLOWERING LOCUS T	FT	*Phaseolus angularis* (82)	1520	2	1	571	947	322	947

#### Genes involved in photosynthesis and energy metabolism

In addition to its biofuel properties, pongamia is also considered as a potential bioenergy tree species, showing high C sequestration efficiency. Of late, the plant is widely used for avenue plantation for tackling rising atmospheric CO_2_. Sequence information of genes involved in photosynthesis and energy metabolism of pongamia is the key to understand the tree C sequestration efficiency, which is usually different from model crop plants. We identified 226 scaffolds corresponding to energy metabolism and photosynthesis (Supplementary Data 8). The scaffolds represented the genes involved in oxidative phosphorylation (79), photosystem (39), and light harvesting complex proteins (16). Furthermore, 47 scaffolds encoded for proteins involved in the Krebs cycle associated with carbon fixation. Another important class was represented by genes involved in nitrogen and sulfur metabolism, which constituted a significant portion detailing about the nitrogen fixation and sulfur utilization in pongamia (Supplementary Data 8). We have analyzed the sequences of the RUBISCO large subunit (rbcL), the small subunit (rbcS), and phosphoenolpyruvate carboxylase (PEP carboxylase), and deduced their evolutionary position with other related plants (Supplementary Figure 5).

#### Karanjin biosynthesis

The seeds of pongamia contain karanjin, pongapin, pongaglabrin, kanjone, pongaglabol, which are all flavonoids. Karanjin, a furanoflavonol, is known to have insecticidal, acaricidal, and other medicinal properties (Jahan et al., 2021; Singh et al., 2021). In the current study, 22 scaffolds encoding for proteins involved in flavonoid biosynthesis were identified (Supplementary Data 9). However, the detailed metabolic insights into karanjin biosynthesis are yet to be characterized in pongamia.

#### Transcription factors

**FIGURE 7:**
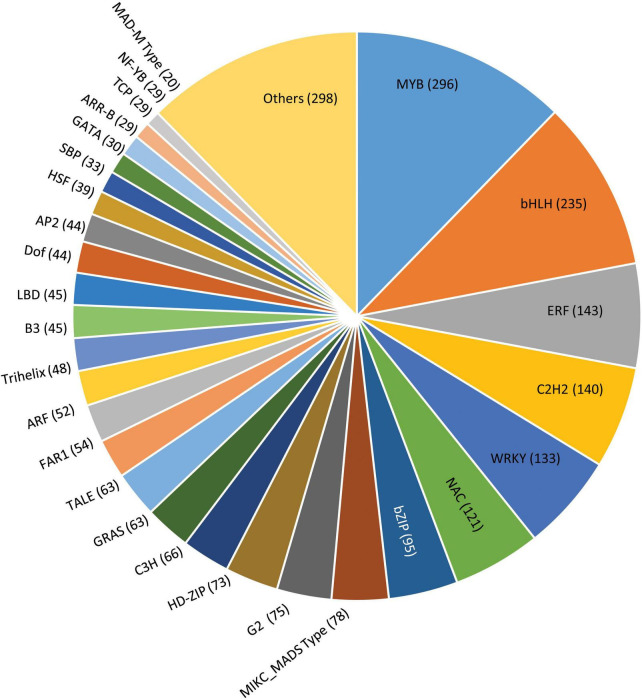
A pie chart showing distribution of scaffolds into various transcription factor families in pongamia. Values in a parenthesis represent the number of scaffolds for each family.

Transcription factors play a key role by binding to the specific elements present on the DNA called DNA-binding domains, thus regulating the gene expression (Strader et al., 2022). In our study, the amino acid sequences from *G. max* were taken as reference for mining the transcription factors of *P. pinnata* wherein a total of 2,373 scaffolds were identified encoding for 52 different types of transcription factor families (Figure 7 and Supplementary Data 10). Among these, the MYB family of proteins constituted a major portion, representing 296 proteins possessing a characteristic MYB DNA-binding domain and are known to play a key role in the plant development, differentiation, defense and metabolism, and abiotic and biotic stresses (Ambawat et al., 2013). Also, MYB-related proteins along with certain other transcription factors like PLATZ, Bhlh, GRAS, G2-like, PHD, CCAAT, can actively participate in regulating the expression of genes involved in fatty acid biosynthesis, elongation, palmitoleate, oleate, and stearate biosynthesis and fatty acid degradation (Troncoso-Ponce et al., 2011; Venglat et al., 2011; Mudalkar et al., 2014), while other transcription factors, such as HY5, ZTL, TCP21, and MYB-related proteins, are involved in regulating circadian rhythms and flowering cycles. Remaining scaffolds fall into the category of other major transcription factors, including bHLH (235) ERF (143), c2h2 (140), WRKY (133), NAC (121), and bZIP (95). A least number of scaffolds were for the RAV family (2) and the S1Fa family (2), while WHIRLY represented by 3 scaffolds followed by NF-X1 (4), LSD (4) HB-PHD (5), GeBP (5), and BBR-BPC (5). Although the transcription factor genes have shown maximum homology with *Glycine max*, there were significant mismatches and non-conserved regions in pongamia, presumably indicating different DNA-binding patterns and gene regulation mechanisms that need a detailed investigation.

## Discussion

*Pongamia pinnata* L. belongs to the Fabaceae family of the Rosids clade and can grow in a variety of agro-climatic regions, making it a potential source of non-edible feedstock for biodiesel production (Scott et al., 2008). To meet the future demands of biofuel, it is critical to identify the elite varieties and establish large-scale plantations of pongamia by extensive selection and propagation (Sunil et al., 2009; Kesari et al., 2010b; Sahoo et al., 2010; Kazakoff et al., 2012). In this study, we report phenotypic characteristics and carbon sequestration potential of five elite pongamia accessions and highlight the bioenergy and biofuel properties of pongamia. Following that, sequencing of the pongamia genome enabled the draft genome assembly of this genetically underexplored tree species in order to better comprehend its complex genomic structure. To the best of our knowledge, this is the first report on the draft genome of a non-model legume tree species, which can act as a potential biofuel feedstock. The sequence information, genome properties, genes, and transcripts identified in this study will answer many questions about inconsistent yields, long gestation periods, flower drop, gall midge disease, stress tolerance, and varietal polymorphism in pongamia.

### Photosynthesis and carbon sequestration

The leaf gas exchange parameters of five pongamia accessions revealed the carbon sequestration potential of pongamia. Lower *P*_*n*_ rates in TOIL424 and TOIL407 when compared to other accessions can be attributed to lower levels of g_*s*_, which might have restricted the supply of CO_2_ to the carboxylation site of Rubisco. It was evident from the data that P_*n*_ rates of both TOIL424 and TOIL407 were decreased with concomitant decrease in g_*s*_, suggesting the stomatal regulation of photosynthesis. There was no change in intercellular CO_2_ (Ci) levels in the accessions, suggesting absence of mesophyll limitation to internal CO_2_ diffusion and also indicating that the reduction of photosynthesis was not solely associated with restriction to CO_2_ in TOIL424 and TOIL407. The decrease in g_*s*_, with no change in the transpiration rate, indicated nonstomatal control of leaf transpiration in pongamia. Higher P_*n*_ rates led to higher leaf-level water use efficiency in TOIL055, TOIL224 compared to TOIL424 and TOIL407, implying that these accessions may tolerate moderate drought stress conditions and give better yields even in stressful environments. It is evident from our data that the P_*n*_ rates also correlated with pod yield of the accessions (Figure 2 and Table 1). Thus, the direct screening of genotypes for higher P_*n*_, g_*s*_, and WUE_*i*_ under natural climatic conditions should be advantageous in selecting the germplasm to attain higher productivity. Photosynthetic CO_2_ assimilation is crucial to obtain feedstock for biofuel production, which includes lignin (for ethanol), cellulose (for bioethanol), starch (for bioethanol), and oils (for biodiesel) (Ranadheer et al., 2019; Antar et al., 2021; Parida et al., 2022). Also, it is the ultimate process that produces ATP and NADPH, which are subsequently utilized in the Calvin cycle and other biochemical pathways to produce the sugars, starch, oils, and other bio-molecules that collectively form biomass. In 5 years of growth, pongamia accessions accumulated an average dry biomass of the 110 kg tree^–1^, which is much higher than other biofuel and bioenergy crops, including jatropha and mulberry (Sekhar et al., 2014, 2015; Kumar et al., 2017). This could be attributed to its higher carbon sequestration capacity wherein the photosynthetic rates were uniformly maintained during 5 years of the growth period, which is unusual for many other tree species. Legume crops and trees fix atmospheric nitrogen through symbiotic association with *Rhizobium* spp., and this could play an important role in maintaining a stoichiometric equilibrium between the N reserves and sequestered C (Sreeharsha et al., 2015). Being a legume tree, pongamia actively formed root nodules as evidenced in this study (Figure 1), and the symbiotic N fixation could presumably be responsible for more biomass accumulation and carbon sequestration. The amount of CO_2_ sequestered per tree by pongamia was relatively more when compared to Jatropha (24 kg carbon per tree) (Kumar et al., 2017), corroborating the fact that pongamia is a dependable tree species for long-term CO_2_ mitigation from the atmosphere in a substantial and accountable manner besides producing biofuel.

Pongamia accessions, including TOIL055, TOIL424, TOIL224, TOIL407, and TOIL620 whose yields ranged from 20.5 to 27.1 kg tree^–1^ year^–1^, are best suited for establishing large-scale plantations for providing ample feedstock, meeting industrial demands. Our further calculations on oil yields showed that TOIL055 has the potential of 67 tons/hectare/year of pod yield, which would approximately yield 5,884 gallons of oil. The pod and seed characteristics of 5 pongamia accessions documented in this study slightly vary with those of previously reported elite varieties (Kaushik et al., 2007; Sunil et al., 2009; Kesari et al., 2010a). The outcrossing reproductive strategy results in wide genetic diversity among pongamia accessions, which manifests in almost all physiological characteristics of the plant (Kesari et al., 2008). Correlating several phenotypic differences in plant height, canopy width, leaf and seed morphology, as well as differences in oil quantity with pod yield, should serve as physical markers to identify elite varieties. Pod yield positively correlated with plant height, canopy width, and the leaf area, and this could be considered as a positive descriptor for selection of elite varieties. The bottom line is that one should consider the plants with more height and canopy size as well as with more seed thickness to obtain higher seed and oil yields to meet the industrial demands. TOIL055, TOIL424, TOIL224, TOIL407, and TOIL620 are such accessions, fulfilling the above criteria, and the germplasm is preserved in our experimental farm for further experimentation *via* establishment of plantations. Seed oil content of the pongamia, which was reported to be in the range of 20 to 38%, is the key feature to categorize the accessions into elite varieties (Dwivedi and Sharma, 2014). The oil quantity of the accessions reported in this study ranged from 28 to 36%, and, interestingly, it had no correlation with pod yield or 100-seed weight. Nevertheless, the total extractable oil is more when the plant produced more pods in the case of the reported elite accessions. The saponification number, iodine value, and cetane number of pongamia FAMEs certainly meet the global biofuel industry standards (Mitra et al., 2021). Other important feature like the cloud point (8.3°C) and the pour point (2.1°C) of pongamia oil are highly satisfactory for its use in tropical and some temperate climates (Viswanathan et al., 2013). In this study, the fatty acid compositions, as revealed by the GC/MS analysis of pongamia seed oil, did not differ significantly among different accessions, as well as from the information in the available literature. Qualitatively, the oil was predominated by oleic acid (55%), followed by linoleic acid (14%), and palmitic acid (8%). However, there needs to be improvement in the seed and oil yields of pongamia by comprehensive understanding and alteration in the seed oil composition through molecular breeding as well as genomic approaches, which is critical to the implementation of biodiesel use in varied climatic conditions as already manifested in other biofuel plants, including camelina and jatropha.

### Genomic elements that govern the growth, development, and productivity in pongamia

Whole genome sequencing can provide thousands of nuclear markers for phylogenetic and population level studies, enabling genome-wide investigations into fundamental aspects. Also, the next generation sequencing technologies and bioinformatics tools enable assembly and annotation of short reads into a meaningful genome, particularly for non-model organisms without a known reference. In the legume family, the genome of several crop plants, including *Cajanus cajan*, *Glycine max*, *Medicago* spp., and *Lotus japonicus*, were successfully sequenced, and the sequence information is available in the public databases (Sato et al., 2008; Schmutz et al., 2010; Young et al., 2011; Varshney et al., 2012). For sequencing the pongamia genome, we have chosen different libraries with varied read lengths to maximize the number of reads and quality of alignment. The overall high-throughput sequencing of three libraries resulted in generating ∼65.2 gb of data, representing approximately ∼60-fold coverage of the predicted *P. pinnata* genome (Choudhury et al., 2014). The assembly statistics, including N50 value, BUSCO groups, number of contigs, and total contig length, demonstrated that the library preparation in this study was optimum to obtain good number of contigs and scaffolds as reported in similar studies (Sato et al., 2008; Young et al., 2011). In general, high N50 value indicates that the assembly contains fewer and lengthy contigs, representing entire chromosome sequence. However, the lower N50 and BUSCOs in this study when compared to assemblies of other plant species can be attributed to the inclusion of more number of contigs with shorter lengths, with the aim of obtaining maximum possible gene fragments with the available short reads. A significant homology of gene sequences obtained in this study with that of transcriptome of pongamia corroborates the fact that the assembly and annotation were of good quality. Nevertheless, the N50 value and BUSCO groups can be further enhanced by filling the gaps with the help of sequencing reads of different lengths and chemistry, for example, minion Nanopore sequencing. Furthermore, there was no significant difference among different sequencing platforms in read utilization percentage and number of reads, thus indicating optimum utilization of sequencing data for valid assembly. In general, the length of contigs, which ranges from 20 to 200 Kb, depends on the read chemistry and quality of raw reads wherein the largest contig in jatropha was reported as 29.74 Kb and as 185.39 Kb in pigeonpea (Sato et al., 2008; Varshney et al., 2012). The size of the assembly of the pongamia draft genome is ∼685 Mb, with ∼0.02% of gaps, which represents 62% of the ∼1,100 Mb of the predicted genome through flow cytometry (Choudhury et al., 2014). There are 54 species belonging to Rosids clade with complete genome sequence available in the public databases, with a maximum size of 1,130 Mb and minimum of 130 Mb, representing *Brassica napus* and *Capsella rubella*, respectively (Slotte et al., 2013; Chalhoub et al., 2014), while, in the Fabaceae family, 9 plant species were sequenced wherein *Glycine max* showed the largest genome size of 1,115 Mb and *Phaseolus vulgaris* had shown a minimum sequenced genome of 520 Mb (Schmutz et al., 2010). We used the BUSCO gene set to validate the genome assembly and found that 65.4% of all BUSCO plant genes were represented in the assembly, providing quantitative measures of the completeness of annotated gene sets in terms of expected gene content (Supplementary Table 3). For genomics data quality control, BUSCO provides a biologically meaningful completeness metric that complements technical measures like N50 of contig or scaffold. It detects duplicated, fragmented, and missing genes, as well as allowing quantitative comparisons with data from model organisms or closely related species (Waterhouse et al., 2018). BUSCO data sets comprise genes evolving under “single-copy control” that is within each lineage they are near-universally present as single-copy orthologs. In pongamia, the presence of fewer missing BUSCOs in the genome assembly and annotated gene sets indicates the sequencing and assembly and annotation captured the complete expected gene content to the maximum. Furthermore, low sequence identity of duplicated BUSCOs in the overall assembly could indicate that the genome annotation successfully gathered sequenced haplotypes. The sequencing depth, quality of assembly, and annotation invariably depend on genome size and complexity, and the predicted genome sizes usually realized only after re-sequencing and reassembly. The draft genome of other important biofuel crop jatropha was reported to be 70–75% of its predicted genome, while it was 72% in the food legume crop, pigeonpea (Sato et al., 2008; Varshney et al., 2012).

Most of the eukaryotic genome consists of repetitive DNA sequences, such as SINE’s, LINE’s, and transposable elements, which are known to be interspersed throughout the chromosome or restricted to centromere or telomeres as junk DNA. Repetitive DNA sequences are also predominant in the plant genome, and they range from 3% of the *U. gibba* genome to 85% of the maize genome (Michael, 2014). They consist of a plethora of genetic information and considered as hotspots for evolution of eukaryotic taxa. Among various categories of repetitive DNA sequences, the transposable elements are capable of moving their locations within the genome so that they can generate genomic plasticity by inducing various chromosomal mutations and allelic diversity. In addition to polyploidization, transposon amplification is also considered as the main mechanism to increase the genome size of a particular species during its evolution (Fedoroff, 2000). Nevertheless, there are certain epigenetic modifications that regulate the uncontrolled proliferation of transposable elements due to which the size and the number of transposons vary from species to species (Lee and Kim, 2014). Well-assembled genome sequences are necessary to characterize different classes of repetitive elements to identify large-scale gene colinearity across related species and to reconstruct the organization and evolution of transposable elements (Bennetzen and Wang, 2014). *De novo* repeat analysis in pigeonpea identified 51.7% of repetitive DNA, whereas *R. communis* and *G. max* had 50 and 59%, respectively (Chan et al., 2010; Schmutz et al., 2010; Varshney et al., 2012). The total number of repeats identified in jatropha was 36.6% of which 29.9% were class I transposable elements, including Gypsy and copia-type LTR retroelements (Sato et al., 2008). Pongamia repetitive DNA observed in the current study is less when compared to other legume species and biofuel tree species, which, presumably, indicate presence of more protein-coding regions or other non-translated regions. Molecular markers, including Restriction Fragment Length Polymorphisms (RFLPs), Randomly Amplified Polymorphic DNA (RAPDs), Single nucleotide polymorphism (SNPs), and microsatellites/SSRs (Simple Sequence Repeats), play an important role in identification of cells, individuals, or species and, also, in the construction of linkage maps, plant breeding, and marker-assisted selections in plants. The genome of jatropha revealed the presence of 41,428 SSRs of which 46.3, 34.3, and 19.4% of di-, tri-, and tetra-nucleotide SSRs, respectively (Sato et al., 2008). In comparison, the Pongamia genome possesses higher number of mono- and tetra- nucleotide SSRs when compared to other repeats, and, also, the study resulted in identification of significantly high number of SSRs (406,385), which could play a major role in marker-assisted breeding in Pongamia for its improvement. Plant species are known to have unique mitochondrial genomes that vary in size and number of genes based on their evolutionary gain or loss. The soybean mitochondrial genome was annotated to 58 functionally known genes and 52 genes with unknown functions (Chang et al., 2013). Similarly, the pongamia mitochondrial genome contains 11 hypothetical proteins as reported in this study.

In this study, we have performed rigorous phylogenetic analysis that provides valuable information about evolutionary conservational status of pongamia. Certainly, it is interesting to know the phylogeny of pongamia, which is a versatile leguminous tree species with other Fabaceae members that are dominated by crop plants of a shorter life cycle. Based on the phylogeny of orthologous clusters, pongamia out-grouped from *G. max* and *Cajanus cajan*, revealing its unique abilities to synthesize oil and other commercially important products like karanjin (Figure 6). In the large Rosids clade, the Fabids group of Eurosids was subdivided into 8 orders where Fabales and Malpighiales form two distinct paraphyletic groups. Although jatropha and pongamia are potential biodiesel-producing plants belonging to Malpighiales and Fabales orders, respectively, it was clear from the data that the fatty acid-producing genes were evolved through independent gene duplication events after the divergence of fabids clade (Supplementary Figure 2A), while another important biofuel plant, such as *Camelina sativa* belonging to Brassicales, out-grouped completely as Malvids diverged from Fabids nearly a 1,000 years ago. The pongamia genome supposedly contains one gene for each enzyme isoform, indicating the absence of obvious gene duplication for lipid metabolism. The degree to which genes remains on corresponding chromosomes (synteny) and in corresponding orders over time varies between eukaryotic genomes (Ghiurcuta and Moret, 2014). Even within close relatives, angiosperm genomes vary dramatically in size and arrangement, with recurring whole-genome duplications accompanied by gene loss that has fragmented ancestral gene linkages across multiple chromosomes over the past 200 million years. Gene synteny and colinearity are explained on the basis of well-conserved sequence blocks (Syntenic blocks) present on one chromosome across the species or genomes. The conservation could be in terms of orientation, adjacency, and position of homologous sequences associated with the corresponding mapped chromosome. Syntenic blocks reduce the complexity of aligning the genomic structures, and they shall be long enough to make their conservation statistically significant. Thus, further elucidation of linkage groups and a genetic map will reveal the conserved syntenic blocks of pongamia to better understand the evolutionary divergence of this potential tree species with other related clades. Pongamia, being an oil seed plant, is important to study the lipid metabolism genes to improve the oil quality for its use as biofuel. Accumulation of unsaturated fatty acids is believed to be an ideal feature for biodiesel production with good cetane number. The FAMEs of pongamia oil belonging to different geographical locations have shown a sizeable portion of oleic acid and linoleic acid as the seed progresses to maturity by the end of the growing season (Pavithra et al., 2012; Singha et al., 2019). The oil biosynthesis is highly regulated temporally through lipid biosynthetic and degradation pathways wherein crucial rate-limiting enzymes are involved. The carboxylation of acetyl CoA to malonyl CoA is the first-rate limiting reaction and is followed by sequential reactions to ultimately generate fatty acyl CoA (Sreeharsha et al., 2016). Our data revealed the structural features along with untranslated regions of key genes, including FAD2, FAD3, and FAD6, as well as PDAT and DGAT, which play an important role in unsaturated fatty acid biosynthesis. Functional characterization of these genes may reveal the unique features of these enzymes, which could help in altering the fatty acid composition of pongamia by modulating the expression patterns. In most tree species, the seed yield and production cycles are dependent on flowering patterns, which, in turn, are regulated by circadian rhythms. Circadian rhythms and plant –pathogen interactions are two crucial mechanisms for pongamia as the inconsistent and lengthy flowering pattern is a hindrance to breeding and cultivation practices. The genes involved in circadian rhythms integrate the environmental signals with internal metabolism and help in the adaptation of plants to different environmental conditions. The sequence information of genes involved in floral transitions, and circadian rhythms will help to modulate the temporal gene expression to alter the flowering cycles in pongamia. Recent studies have postulated important circadian clock genes (ELF4, PRR7, TOC1, and LCL1) in pongamia, which are under diurnal regulation of the central oscillator (Winarto et al., 2015). Our data mined the complete gene sequence of PRR and ELF gene families wherein PRR5 showed more number of introns and possessed longer gene length of 5,743 bp. PRR1, PRR3, PRR5, PRR7, and PRR9 are members of the PRR gene family and have important roles in the central oscillator, whereas ELF3 and ELF4 are clock genes known to form an evening complex (EC) with LUX (Doyle et al., 2002; Khanna et al., 2003; Nozue et al., 2007; Nusinow et al., 2011). The complex synchronization between these clock genes generates circadian rhythms, thus regulating the output pathways, such as flowering. LHY and MYB75 are MYB-like transcription factors that play pivotal roles in the morning loop of the central oscillator. Since the MYB domain is known for DNA binding, these transcription factors could play an important role in the DNA-binding activity of pongamia LCL1 (Carré and Kim, 2002). The genes that show circadian rhythms will have a role in regulating the grain yield, grain weight, number of grains per panicle, and flowering time in many plants, including legumes. The plant’s physiological phenomenon, such as flower opening, nectar secretion, seed composition, and development, was controlled by genes that are under circadian control (Hudson, 2010; Preuss et al., 2012). The regulatory networks of these genes can be edited using the sequence information deduced in this study, which will have a significant impact on modulating the flowering cycles in pongamia.

## Conclusion

Our data present crucial physiological and morphological parameters to establish agroforestry carbon projects using pongamia. We have identified physiological descriptors as well as genes that control seed yield potential in pongamia accessions that help in huge plantations. Furthermore, we sequenced the whole genome of *Pongamia pinnata* to unravel the sequence information of genes involved in important metabolic pathways. We report ∼680 Mb of a genome sequence, which is ∼62% of the predicted 1.1 Gb of the pongamia genome. The sequencing strategy of including multiple Illumina sequencing platforms enhanced the sequencing depth and quality of raw reads, thus facilitating low-cost genome sequencing. A complete set of annotated gene sets offers a starting point for a detailed characterization of gene functions, biochemical and regulatory pathways or quantitative trait loci, thus facilitating comparative genomics and evolutionary studies. The current pongamia assembly provides a useful reference for future efforts to establish a complete genome for this potential tree species. We deduced characteristics of genes involved in crucial metabolic pathways of pongamia, including fatty acid metabolism, energy metabolism, flowering regulation, and karanjin biosynthesis. The phylogenetic analysis showed the evolutionary conservation of pongamia genes and the position of pongamia with respect to other related species. Our data are the first step toward characterizing the full genome of this potential biofuel-yielding tree species. Further attempts of re-sequencing with different read chemistry will certainly improve the genetic resources at a chromosome level and accelerate the molecular breeding programs for pongamia.

## Data availability statement

The genome assembly data, annotations, and gene models were submitted to the NCBI Sequence Read Archive (SRA) and BioSample databases with BioSample accession number SAMN12217327 and BioProject ID PRJNA552760. The SRA accessions of three libraries are SRX6412242, SRX6412241, and SRX6412240.

## Author contributions

RS, SM, and AR participated in designing experiments, analyzed the data and discussed results, and wrote the manuscript. RS and SM performed the experiments. All authors contributed to the article and approved the submitted version.

## Footnotes

1
http://busco.ezlab.org
