# Supplementary material for: Genome sequencing and analysis uncover the regulatory elements involved in the development and oil biosynthesis of Pongamia pinnata (L.) – A potential biodiesel feedstock
Source: Front Plant Sci. 2022 Aug 25;13:747783. doi: 10.3389/fpls.2022.747783 (PMC9454018; doi:10.3389/fpls.2022.747783)
Supplement: Supplementary file 1 [file Table_1.pdf]

Supplementary Table 1: Pearson coefficient matrix showing correlation coefficient (r^2) between 14 morphological and reproductive traits among 5 Pongamia accessions. Also represented the corresponding significance of correlation using *'p'* values in shaded cells. Values represented in bold indicate significant correlation.

	Plant Height	Canopy Width	Plant girth	Leaf length	Leaf width	Pod length	Pod width	100-Pod wt	Seed length	Seed width	Seed thickness	100-Seed wt	Pod yield	Oil content
Plant Height	**1**	**0.019**	0.333	0.455	0.310	0.444	0.919	0.643	**0.002**	0.455	0.160	**0.033**	**0.045**	0.593
Canopy Width	**0.355**	**1**	0.164	0.310	0.031	0.375	0.229	0.227	0.139	0.921	0.213	0.102	**0.001**	0.645
Plant girth	0.072	0.143	**1**	0.649	0.371	0.953	0.815	0.505	0.764	0.650	0.102	**0.028**	0.236	0.299
Leaf length	0.044	0.079	0.016	**1**	**0.000**	0.821	0.146	0.071	0.693	0.501	0.828	0.729	**0.047**	0.095
Leaf width	0.079	**0.312**	0.062	**0.622**	**1**	0.932	0.329	0.199	0.840	0.278	0.617	0.684	**0.006**	0.479
Pod length	0.046	0.061	0.000	0.004	0.001	**1**	0.478	0.795	0.935	0.322	**0.006**	0.141	0.475	0.326
Pod width	0.001	0.109	0.004	0.155	0.073	0.039	**1**	**0.001**	0.321	0.252	0.520	0.827	0.290	0.273
100-Pod wt	0.017	0.110	0.035	0.229	0.123	0.005	**0.713**	**1**	0.506	0.894	0.613	0.750	0.276	0.333
Seed length	**0.548**	0.161	0.007	0.012	0.003	0.001	0.076	0.035	**1**	0.372	0.448	0.470	0.238	0.485
Seed width	0.044	0.001	0.016	0.036	0.090	0.075	0.100	0.001	0.062	**1**	0.201	0.438	0.475	0.889
Seed thickness	0.146	0.117	0.193	0.004	0.020	**0.458**	0.032	0.020	0.045	0.123	**1**	**0.002**	0.312	0.760
100-Seed wt	**0.582**	0.192	**0.321**	0.010	0.013	0.159	0.004	0.008	0.041	0.047	**0.526**	**1**	0.254	0.713
Pod yield	**0.411**	**0.783**	0.106	**0.420**	**0.449**	0.040	0.086	0.090	0.105	0.040	0.078	0.099	**1**	0.988
Oil content	0.023	0.017	0.082	0.199	0.039	0.074	0.091	0.072	0.038	0.002	0.007	0.011	0.000	**1**

wt = weight
