# Supplementary material for: Genome sequencing and analysis uncover the regulatory elements involved in the development and oil biosynthesis of Pongamia pinnata (L.) – A potential biodiesel feedstock
Source: Front Plant Sci. 2022 Aug 25;13:747783. doi: 10.3389/fpls.2022.747783 (PMC9454018; doi:10.3389/fpls.2022.747783)
Supplement: Supplementary file 2 [file Table_2.pdf]

Supplementary Table 2: The number of reads generated from three different libraries and sequencing platforms.

Sample Library	Raw Reads	Processed Reads
HiSeq	80818900	75228000
MiSeq	19983452	17703080
NextSeq	103336716	81012606
**Total**	**204139068**	**173943686**
