# Supplementary material for: Genome sequencing and analysis uncover the regulatory elements involved in the development and oil biosynthesis of Pongamia pinnata (L.) – A potential biodiesel feedstock
Source: Front Plant Sci. 2022 Aug 25;13:747783. doi: 10.3389/fpls.2022.747783 (PMC9454018; doi:10.3389/fpls.2022.747783)
Supplement: Supplementary file 3 [file Table_3.pdf]

Supplementary Table 3: Percentage of different classes of BUSCOs from the genome assembly of *Pongamia pinnata*.

Complete BUSCOs (C)	3499
Complete and single-copy BUSCOs (S)	3396
Complete and duplicated BUSCOs (D)	103
Fragmented BUSCOs (F)	457
Missing BUSCOs (M)	1410
Total BUSCO groups searched	5366
C:65.2%[S:63.3%,D:1.9%],F:8.5%,M:26.3%,n:5366	
