# Supplementary material for: Genome sequencing and analysis uncover the regulatory elements involved in the development and oil biosynthesis of Pongamia pinnata (L.) – A potential biodiesel feedstock
Source: Front Plant Sci. 2022 Aug 25;13:747783. doi: 10.3389/fpls.2022.747783 (PMC9454018; doi:10.3389/fpls.2022.747783)
Supplement: Supplementary file 4 [file Table_4.pdf]

Supplementary Table 4: The statistics for different SSR markers obtained in Pongamia genome.

SSR Statistics	Number
Total number of sequences examined	199825
Total size of examined sequences (bp)	685887678
Total number of identified SSRs	406385
Number of SSR containing sequences	143942
Number of sequences containing more than 1 SSR	100447
Number of compound SSRs	77879
Mono nucleotide repeat (p1)	260290
Di nucleotide repeat (p2)	85213
Tri nucleotide repeat (p3)	35732
Tetra nucleotide repeat (p4)	100805
Penta nucleotide repeat (p5)	1538
Hexa nucleotide repeat (p6)	686
