# Supplementary material for: Genome sequencing and analysis uncover the regulatory elements involved in the development and oil biosynthesis of Pongamia pinnata (L.) – A potential biodiesel feedstock
Source: Front Plant Sci. 2022 Aug 25;13:747783. doi: 10.3389/fpls.2022.747783 (PMC9454018; doi:10.3389/fpls.2022.747783)

Supplementary Figure 1: Distribution of scaffolds into various classes of metabolic pathways based on the KAAS analysis indicating the percentage of distribution.

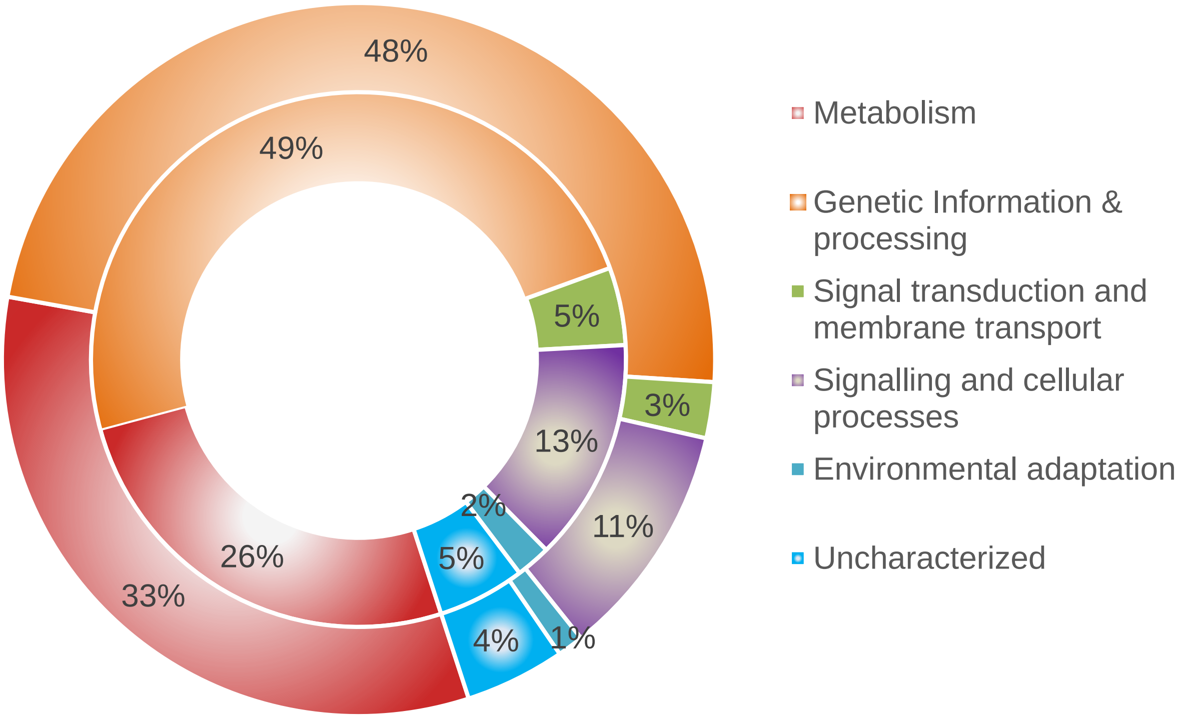

Supplementary Figure 2: The phylogenetic relationship of Pongamia with other related organisms based on the orthologous proteins of A) energy metabolism B) plant-environment interaction C) lipid metabolism D) transcription factors. Seaview program was used to compute the phylogenetic tree. The program used a fast distance-based (Neighbor-Joining) method, BioNJ, to compute a full initial tree. The values present in the image represent the branch length.

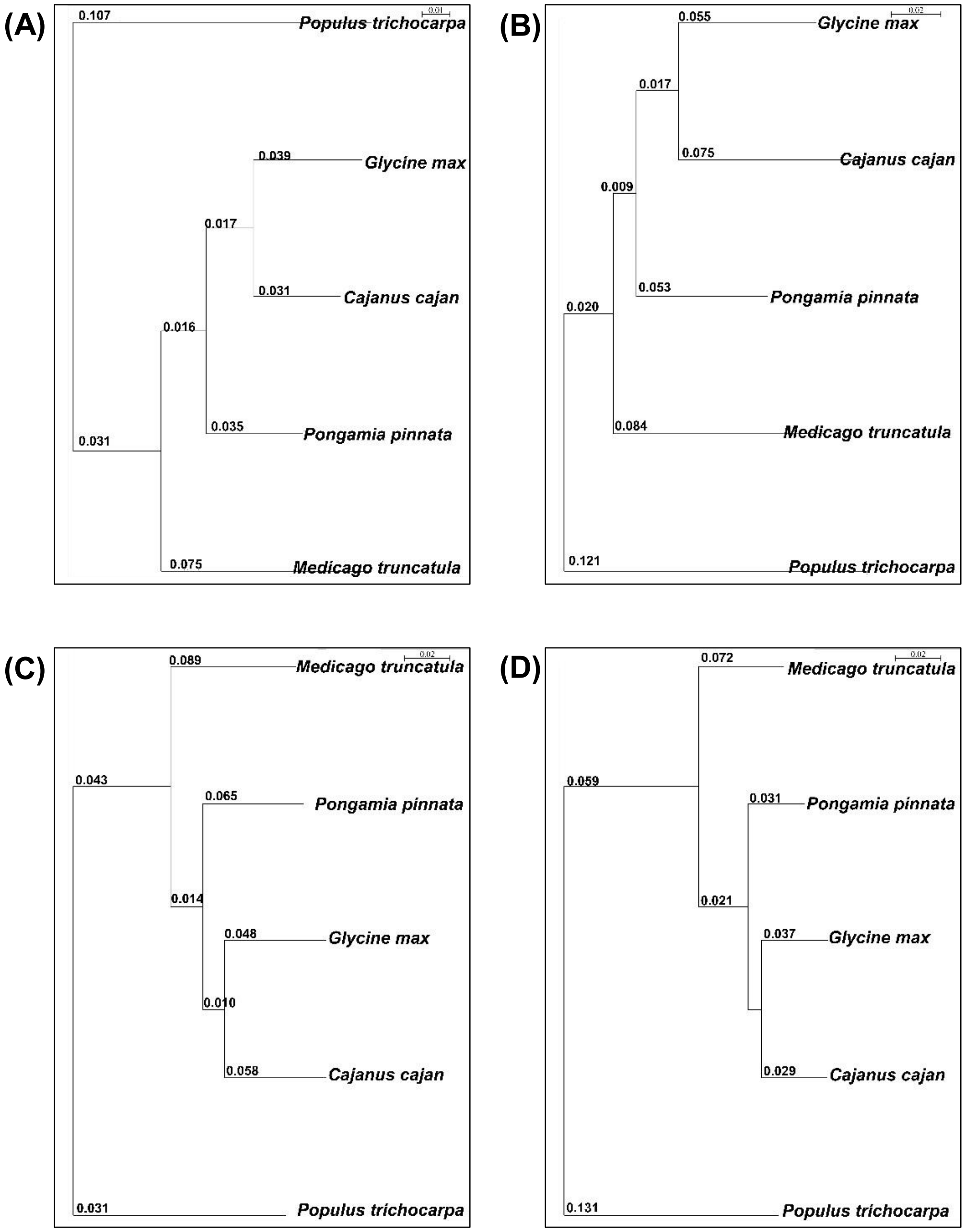

Supplementary Figure 3: Pongamia lipid biosynthetic genes and their phylogenetic relationship with ortholog proteins from other related organisms. The evolutionary history was inferred using the Neighbor-Joining method. The bootstrap consensus tree inferred from 1000 replicates is taken to represent the evolutionary history of the taxa analyzed. Branches corresponding to partitions reproduced in less than 50% bootstrap replicates are collapsed. The evolutionary distances were computed using the JTT matrix-based method and are in the units of the number of amino acid substitutions per site. All positions containing gaps and missing data were eliminated. Evolutionary analysis were conducted in MEGA7.

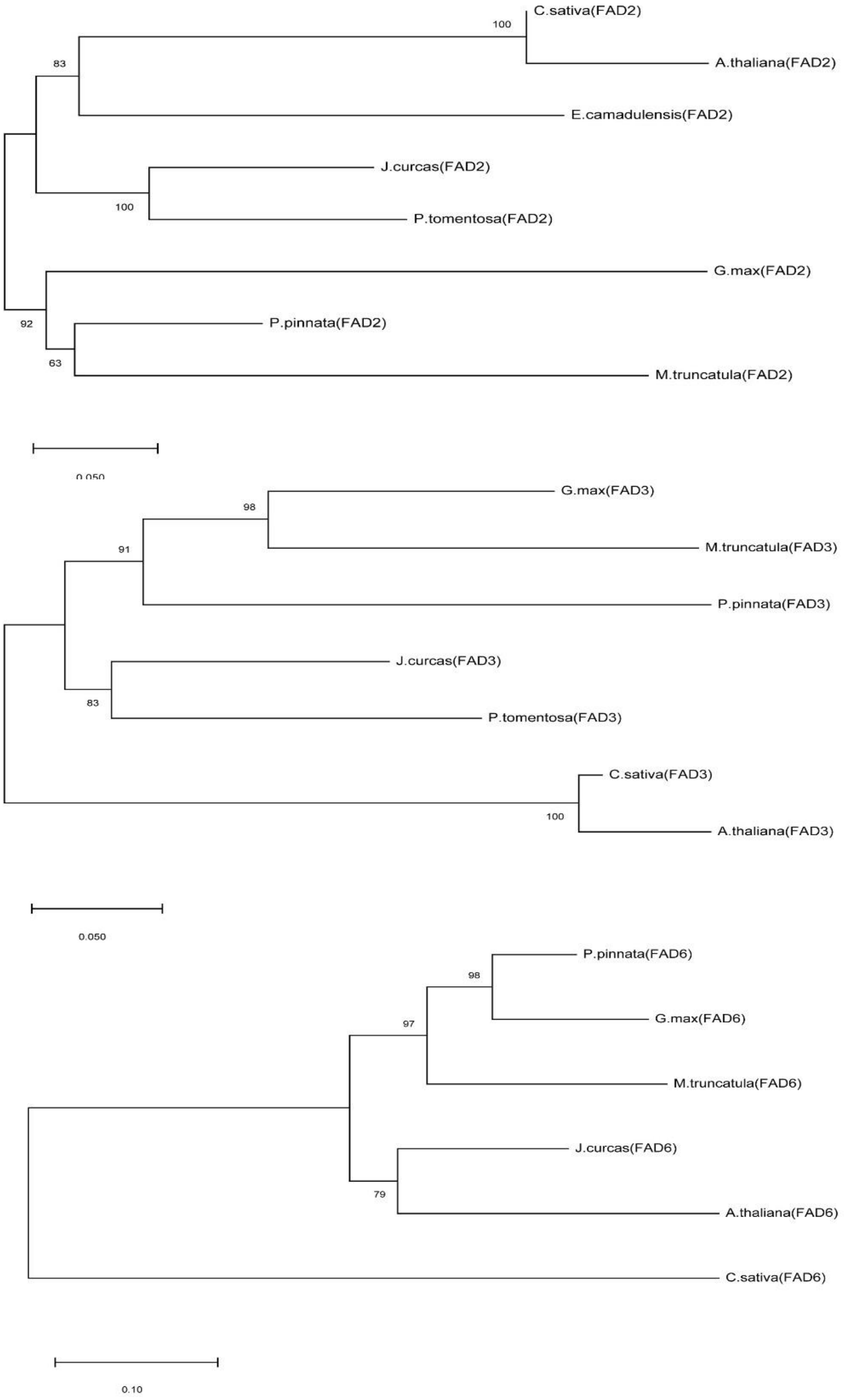

Supplementary Figure 4: Pongamia flowering related genes and their phylogenetic relationship with ortholog proteins from other related organisms. The evolutionary history was inferred using the Neighbor-Joining method. The bootstrap consensus tree inferred from 1000 replicates is taken to represent the evolutionary history of the taxa analyzed. Branches corresponding to partitions reproduced in less than 50% bootstrap replicates are collapsed. The evolutionary distances were computed using the JTT matrix-based method and are in the units of the number of amino acid substitutions per site. All positions containing gaps and missing data were eliminated. Evolutionary analysis were conducted in MEGA7

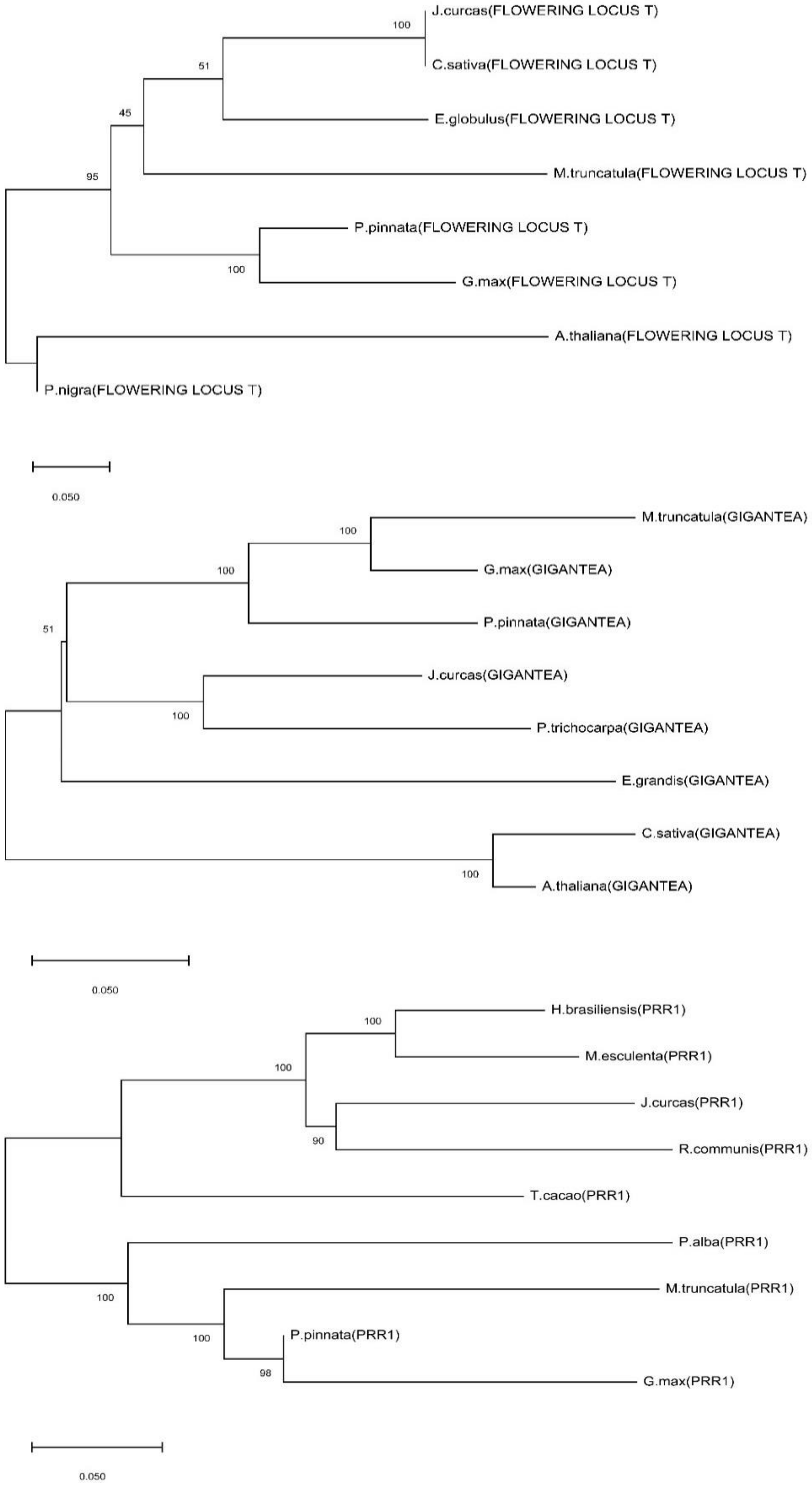

Supplementary Figure 5: Pongamia energy metabolism genes and their phylogenetic relationship with ortholog proteins from other related organisms. The evolutionary history was inferred using the Neighbor-Joining method. The bootstrap consensus tree inferred from 1000 replicates is taken to represent the evolutionary history of the taxa analyzed. Branches corresponding to partitions reproduced in less than 50% bootstrap replicates are collapsed. The evolutionary distances were computed using the JTT matrix-based method and are in the units of the number of amino acid substitutions per site. All positions containing gaps and missing data were eliminated. Evolutionary analysis were conducted in MEGA 7.

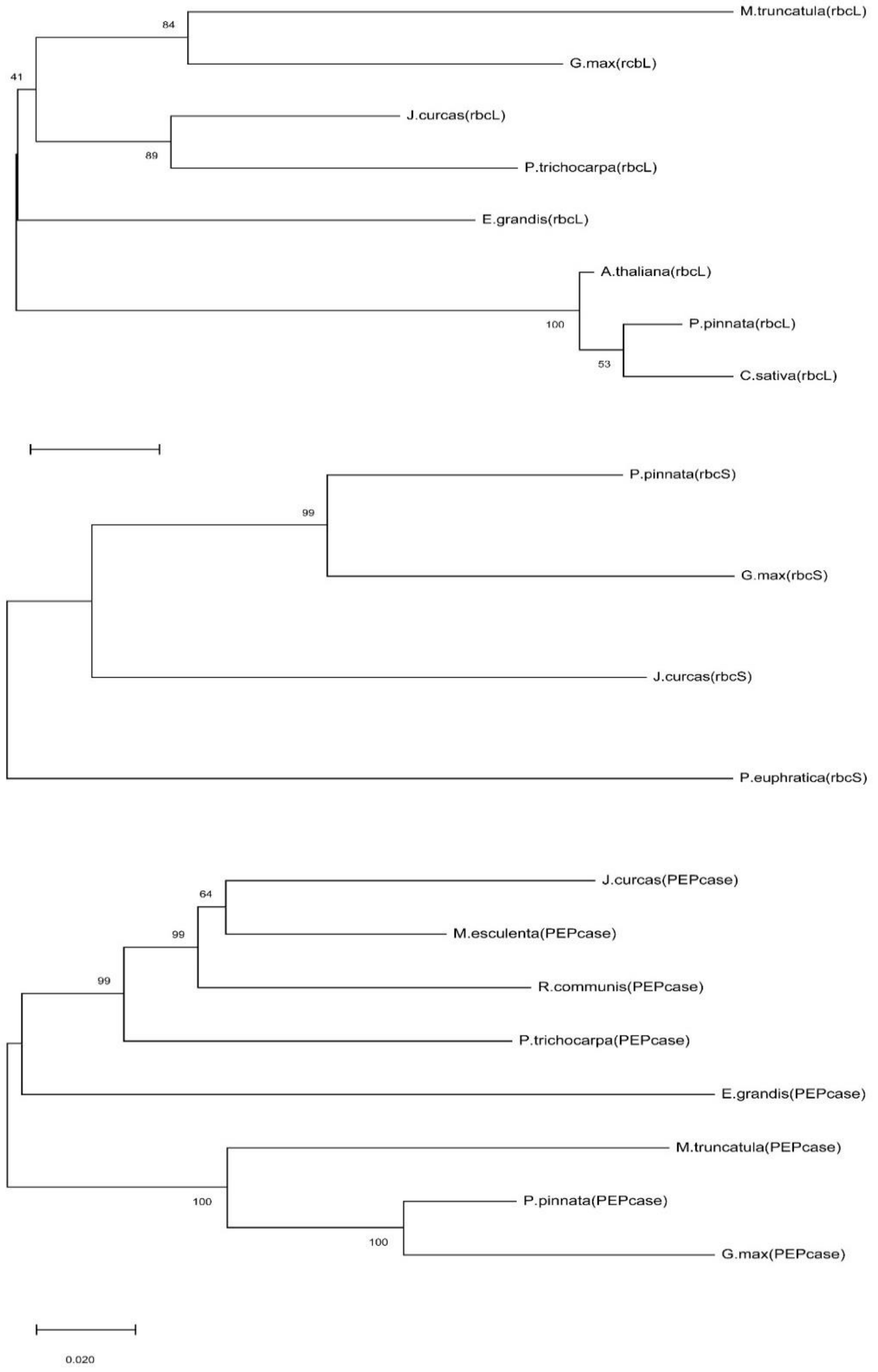

Supplementary Figure 6: Pictorial representation of block planting design followed in the experimental field to grow different accessions of *Pongamia pinnata*

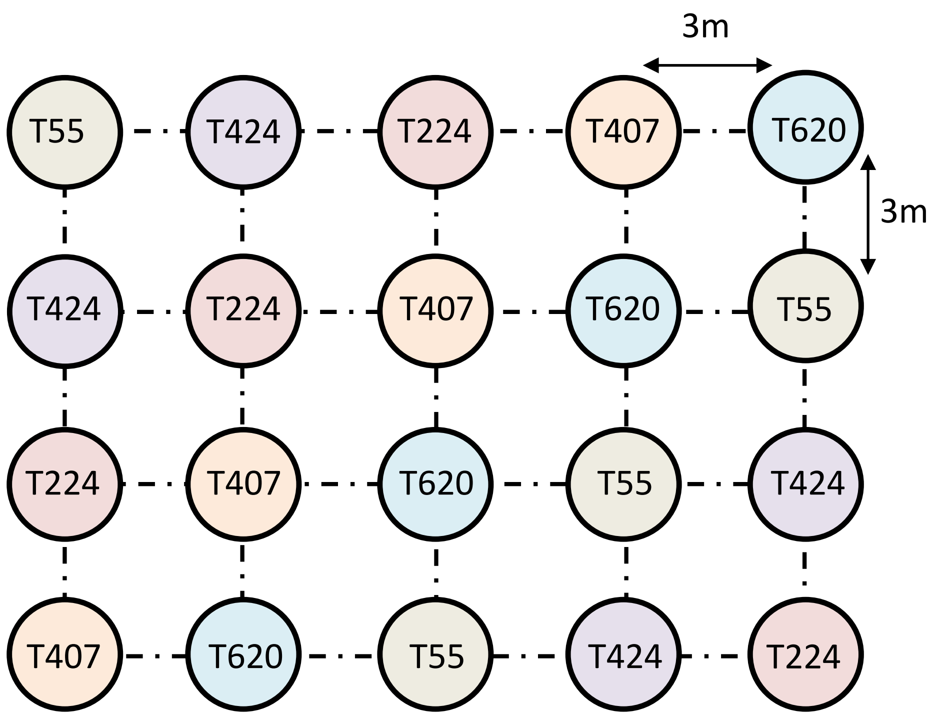

Supplement: Supplementary file 15 [file Data_Sheet_11.pdf]
